# Supplementary material for: Quantitative magnetic resonance cholangiopancreatography metrics improve prognostication in primary sclerosing cholangitis
Source: JHEP Rep. 2026 May 12;8(8):101892. doi: 10.1016/j.jhepr.2026.101892 (PMC13320491; doi:10.1016/j.jhepr.2026.101892)
Supplement: Multimedia component 1 [file mmc1.pdf]

Quantitative magnetic resonance cholangiopancreatography metrics improve prognostication in primary sclerosing cholangitis

Tim E. Middelburg, Laura Cristoferi, Willemijn Ponsioen, Maud Turkenburg, Carlos Ferreira, Tom Davis, Karin Horsthuis, Ynte S. de Boer, Adriaan J van der Meer, Annemarie C. de Vries, Roy S. Dwarkasing, Johannes A. Bogaards, Sarah Al-Shakhshir, Palak Trivedi, Daphne D'Amato, Mauro Vigano, Eugenia V Pesatori, Cesare Maino, Marco Carbone, Michael Pavlides, Jaap Stoker, Emma L. Culver, Cyriel Y Ponsioen

Table of contents

Fig. S1 2
Fig. S2 3
Fig. S3 4
Fig. S4 5
Fig. S5 6
Fig. S6 7
Table S1 8
Table S2 9
Table S3 10
Table S4 11
Table S5 12
Tabel S6 12
Table S7 14
Table S8 15

Fig. S1 - Time dependent Area under the Curve for event-free survival by qmAOM score over 10 years time. Each line portrays the corresponding year.

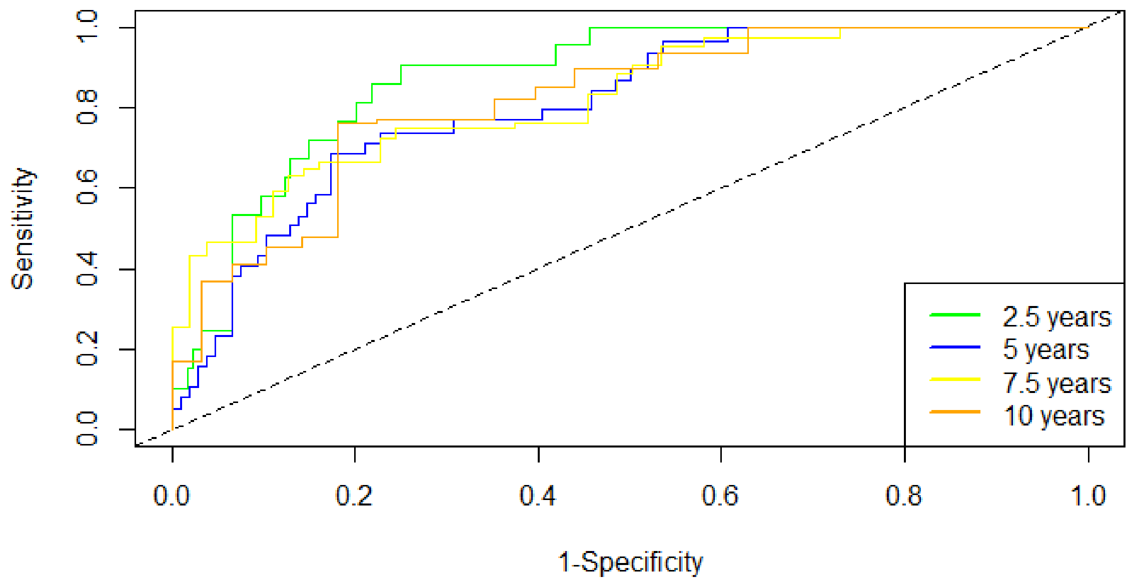

Time (years)	Cases	Survivors	Censored	AUC (%)
2.5	21	195	8	88.2%
5.0	38	114	72	81.6%
7.5	51	64	109	84.2%
10.0	61	122	141	83.6%

Fig. S2 - Kaplan-Meier curve of predicted survival versus observed survival over 10 years time. The black line portrays the observed survival, the blue line portrays the predicted survival.

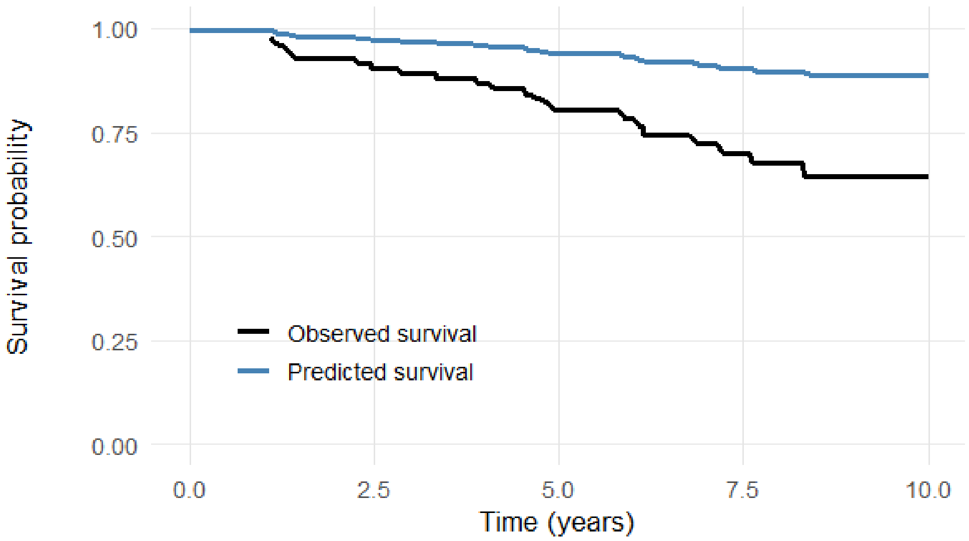

Events	0	9	16	21	23
Censored	0	6	35	44	65
At risk	96	81	45	31	8

Fig. S3 - Kaplan-Meier curves risk groups based on the qmAOM score in the ANALI subset. Based on ≤ 1.29 as low-risk and > 1.29 as high-risk group.

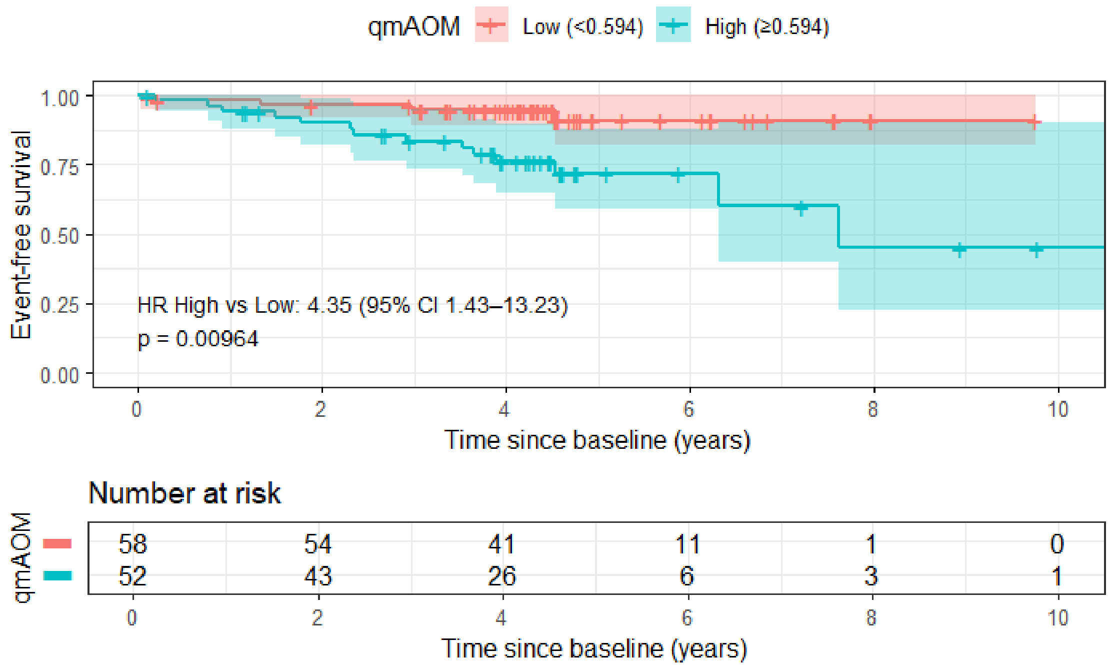

Fig. S4 - Kaplan-Meier curves of risk groups based on the total score of ANALI without gadolinium in the ANALI subset, including reviewer from Monza/Bergamo and reviewer 1 from Oxford. Based on ≤2 as low-risk and >2 as high-risk group.

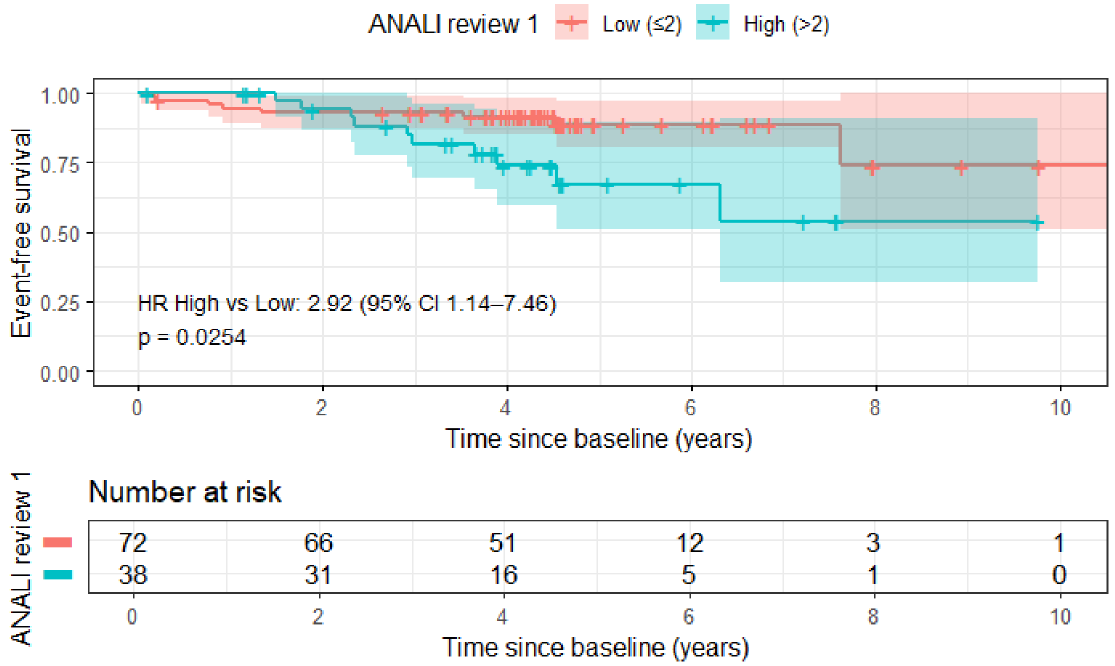

Fig. S5 - Kaplan-Meier curves of risk groups based on the total score of ANALI without gadolinium in the ANALI subset, including reviewer from Monza/Bergamo and reviewer 2 from Oxford. Based on ≤2 as low-risk and >2 as high-risk group.

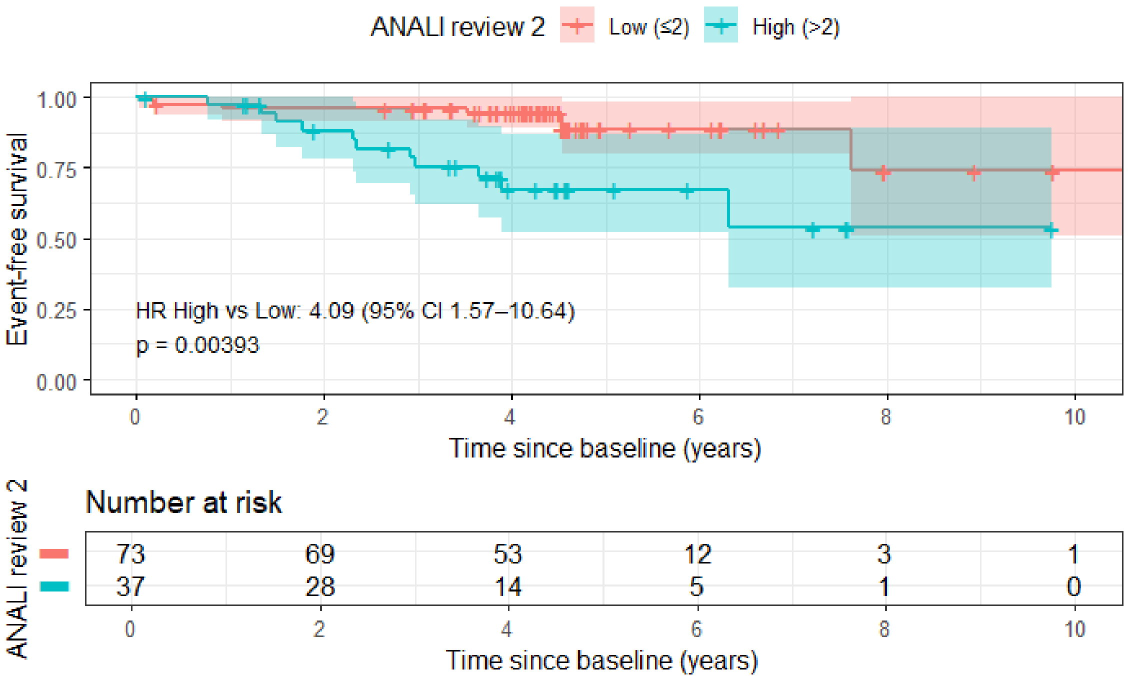

Fig. S6 - Heatmap of significant correlations between MRCP+ metrics, qmAOM and ANALI without gadolinium in the ANALI subset. Including components

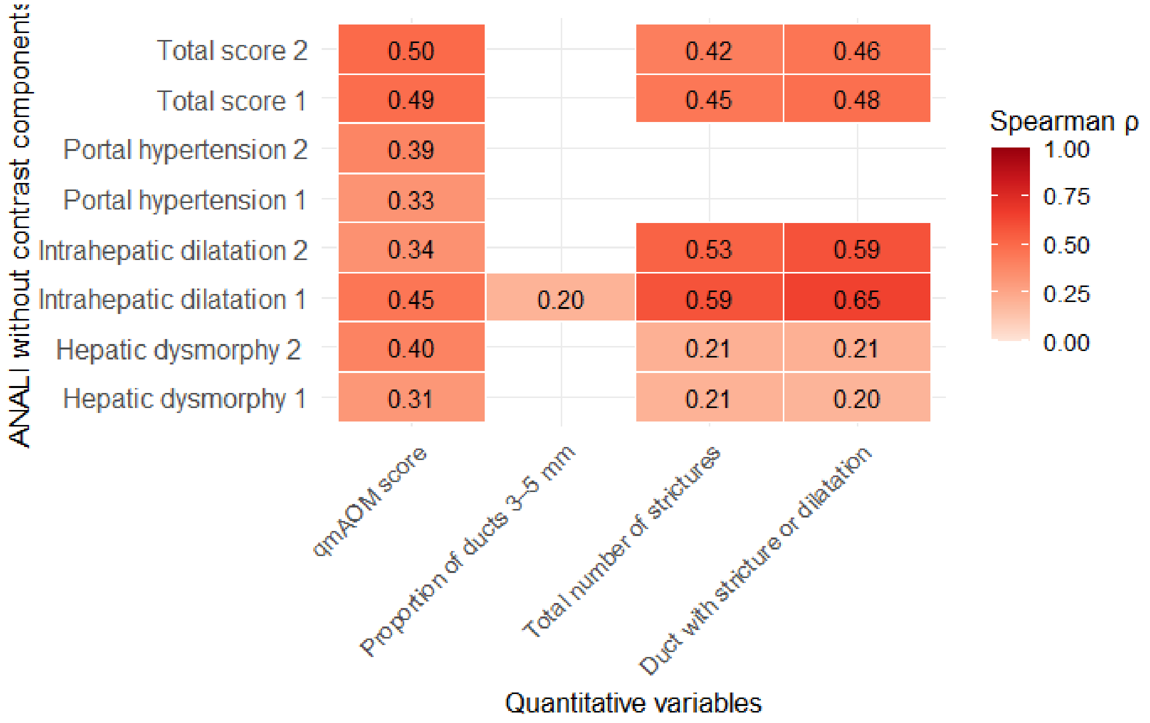

Table S1 - Cohort designs per participating centre

	Centre			
	Monza & Bergamo	***Birmingham***	***Oxford***	***Amsterdam & Rotterdam***
Study design	Retrospective	Prospective	Prospective	Retrospective
Minimum age of participants	> 16 years	> 18 years	> 18 years	> 18 years
PSC definition	EASL	UK-PSC, EASL	AASLD	IPSCSG
Minimal follow-up required	> 3 months	Not applicable	Not applicable	> 1 year
Fasting protocol	6-hours	4-hours	4-hours	Not specified*
Tesla strength	1.5T & 3T	3T	3T	1.5T & 3T

** Details on the fasting protocols were not retrievable, but routinely 4 hr fasting is prescribed before scan acquisition in the Netherlands*

Table S2 – MRI parameters per participants centre

Parameter	Centre			
	Monza & Bergamo	***Birmingham***	***Oxford***	***Amsterdam & Rotterdam***
Sequence	3D multi-shot fast/turbo spin echo	3D multi-shot fast/turbo spin echo	3D multi-shot fast/turbo spin echo	3D multi-shot fast/turbo spin echo
Plane	Coronal	Coronal	Coronal	Coronal
Triggering	Free breathing, navigator based	Free breathing, navigator based	Free breathing, navigator based	Free breathing, navigator based
Number of slices	72-192	72	60	40-240
Slice thickness, mm	1-2.5	1.1	1.1	1 – 2.20
Echo time (TE), ms	505-1144	708	604	441-1182
Repetition time (TR), ms	Breathing cycle	Breathing cycle	Breathing cycle	Breathing cycle
Acquisition matrix	256-416 x 200-356	258 x 320	256 x 256	236-448 x 181x387
FOV, mm	260-420 x 260-420	400x400	280	250-450 x 250-450
Flip angle (degrees)	90-140	120	120	90-140
iPAT factor	Yes	Yes	Yes	Yes
Number of averages	0.5-2	2	1.4	1-2
Fat suppression	Yes	Yes	Yes	Yes

Table S3 - Variable selection frequency by LASSO analysis in bootstrap framework

Variable	Selection frequency (%)
Serum albumin	99.6%
Serum total bilirubin	99.4%
Platelets	94.6%
Percentage of ducts with a 3-5mm median diameter	91.8%
Serum ALP	90.2%
IBD (yes/no)	70.4%
Centre type (transplantation/tertiary)	68.4%
Number of ducts with strictures or dilatations	62.8%
Age at PSC diagnosis	54.8%
Serum AST	52.2%
Total number of strictures	51.6%
Gender	46.6%
Sum of stricture length	42.8%
Median duct diameter	36.6%
Time from diagnosis to MRCP	35.2%
Serum ALT	27.4%
Age at MRCP, y	23.8%

mm: millimetre: ALP: Alkaline Phosphatase; IBD: inflammatory bowel disease; PSC: Primary sclerosing cholangitis; AST: Aspartate aminotransferase; MRCP: Magnetic resonance cholangiopancreatography; ALT: Alanine Aminotransferase

AST, ALP and total bilirubin are expressed in x the ULN and transformed to log-scale; Albumin and platelets are expressed as x the LLN and transformed to log-scale.

Table S4 - Patient demographics per centre type

		Tertiary care centre<br>*N=225*	Transplant centre<br>*N=232*	Overall<br>*N=457*
**Sex**	*Male*	145 (64.4%)	149 (64.2%)	294 (64.3%)
**Age at PSC diagnosis**, *y*		35.9 [26.1, 47.1]	36.8 [24.3, 47.9]	36.2 [25.2, 47.6]
**Type of PSC**	*Large Duct*	203 (90.2%)	223 (96.1%)	426 (93.2%)
	Small Duct	22 (9.8%)	9 (3.9%)	31 (6.8%)
**Type of IBD**	*None*	97 (43.1%)	99 (42.7%)	196 (42.9%)
	UC	113 (50.2%)	115 (49.6%)	228 (49.9%)
	CD	8 (3.6%)	17 (7.3%)	25 (5.5%)
	IBDu	7 (3.1%)	1 (0.4%)	8 (1.8%)
**Age at MRCP+**, *y*		44.0 [32.0, 54.0]	46.0 [29.8, 55.0]	45.0 [31.0, 54.0]
**PSC diagnosis to MRCP+**, *y*		6.0 [0.6, 11.3]	5.2 [2.0, 10.0]	5.6 [1.1, 10.6]
**MRCP+ to event or censoring**, *y*		4.8 [4.2, 8.5]	5.0 [2.2, 7.7]	4.9 [3.4, 8.0]
**PSC diagnosis to last follow-up**, *y*		12.0 [7.7, 17.5]	10.8 [6.8, 15.9]	11.4 [7.1, 16.6]
**ALP (U/L)**, *n = 429*		184 [115, 314]	215 [104, 368]	201 [111, 348]
**Bilirubin (umol/L)**, *n = 418*		13 [9, 21]	15 [10, 32]	14 [9, 25]
**Albumin (g/L)**, *n = 386*		41 [38, 44]$^{+}$	43 [39, 46]$^{+}$	42 [38, 45]
**AST (U/L)**, *n = 430*		45 [29, 86]	52 [31, 86]	49 [30, 86]
**Platelets (x10^9/L)**, *n = 414*		264 [196, 329]	245 [192, 299]	254 [194, 315]
**AOM risk score**, *n = 364*		1.6 [1.2, 2.0]	1.6 [1.2, 2.3]	1.6 [1.2, 2.1]
**AOM low risk (< 2.00)**, *n = 364*		139 (73.5%)	118 (67%)	257 (56.2%)
**Events**	***Primary endpoint****			
	Hepatic decompensation	44 (19.6%)	40 (17.2%)	84 (18.4%)
	Liver transplantation	13 (5.8%)	19 (8.2%)	22 (4.8%)
	PSCR-death exl. CRC	8 (3.6%)	25 (10.8%)	33 (7.2%)
	Secondary endpoints			
	PSCR-death or LT incl. CRC	48 (21.3%)	75 (32.3%)	123 (26.9%)
	PSCR-death or LT excl. CRC	48 (21.3%)	74 (32.9%)	122 (26.7%)

PSC: Primary sclerosing cholangitis; y: years; IBD: inflammatory bowel disease; UC; Ulcerative colitis; CD: Crohn's Disease: IBDu: unspecified IBD; MRCP: Magnetic resonance cholangiopancreatography; ALP: Alkaline Phosphatase; n: number of participants; AST: Aspartate aminotransferase; AOM: Amsterdam Oxford Model. PSCR: PSC-related. LT: Liver transplantation.

Reference ranges: ALP ≤ 120 U/L; Bilirubin ≤ 18 umol/L; Albumin ≥ 35 g/L; AST ≤ 35 U/L; Platelets ≥ 150 x 10^9/L.

Continuous numbers are displayed as total (%) or median [Q1, Q3]

** The composite primary endpoint shows which event occurred first in follow-up after MRCP+.*

$^{+}$ p-value < 0.01. The rest was non-significant.

Tabel S5 - Demographics in the derivation and validation set

		Derivation *N=224*	Validation *N=96*	Overall *N=320*
Sex	*Male*	147 (65.6%)	60 (62.5%)	207 (64.7%)
Age at PSC diagnosis, *y*		35.0 [23.5, 47.0]	35.5 [26.7, 48.3]	35.0 [24.6, 47.4]
Type of PSC	*Large Duct*	210 (93.8%)	89 (92.7%)	299 (93.4%)
	Small Duct	14 (6.3%)	7 (7.3%)	21 (6.6%)
Type of IBD	*None*	87 (38.8%)	49 (51.0%)	136 (42.5%)
	UC	116 (51.8%)	44 (45.8%)	160 (50.0%)
	CD	15 (6.7%)	2 (2.1%)	17 (5.3%)
	IBDu	6 (2.7%)	1 (1.0%)	7 (2.2%)
Age at MRCP+, *y*		44.0 [31.8, 54.0]	47.0 [31.0, 56.0]	44.5 [31.0, 54.2]
PSC diagnosis to MRCP+, *y*		5.9 [2.0, 10.6]	4.5 [1.3, 11.4]	5.7 [1.5, 11.2]
MRCP+ to event or censoring, *y*		5.3 [4.1, 7.8]	4.8 [3.9, 8.1]	5.1 [4.0, 7.9]
PSC diagnosis to last follow-up, *y*		11.9 [7.8, 16.8]	11.4 [7.1, 15.9]	11.5 [7.7, 16.5]
ALP (U/L)		193 [113, 311]	163 [96, 355]	184 [107, 324]
Bilirubin (umol/L)		13 [9, 21]	13 [9, 24]	13 [9, 21]
Albumin (g/L)		42 [39, 46]	42 [39, 45]	42 [39, 45]
AST (U/L)		48 [30, 81]	42 [27, 77]	46 [29, 81]
Platelets (x10^9/L)		255 [193, 314]	255 [190, 308]	255 [193, 314]
AOM risk score		1.5 [1.2, 2.0]	1.5 [1.2, 2.0]	1.5 [1.2, 2.0]
AOM low risk (< 2.00)		58 (25.9%)	25 (26.0%)	83 (25.9%)
Event	*Primary endpoint*	64 (28.6%)	24 (25%)	88 (27.5%)

PSC: Primary sclerosing cholangitis; y: years; IBD: inflammatory bowel disease; UC; Ulcerative colitis; CD: Crohn's Disease: IBDu: unspecified IBD; MRCP: Magnetic resonance cholangiopancreatography; ALP: Alkaline Phosphatase; n: number of participants; AST: Aspartate aminotransferase; AOM: Amsterdam Oxford Model. Reference ranges: ALP ≤ 120 U/L; Bilirubin ≤ 18 umol/L; Albumin ≥ 35 g/L; AST ≤ 35 U/L; Platelets ≥ 150 x 10^9/L. Continuous numbers are displayed as total (%) or median [Q1, Q3]

No significant differences were found between the derivation and validation set.

Table S6 – Observed versus predicted survival and corresponding Brier score in the validation set.

	Year			
	**2.5**	**5**	**7.5**	**10**
**Observed survival**	91%	81%	70%	64%
**Mean predicted survival**	97%	94%	90%	89%
**Brier score**	0.06	0.11	0.14	0.14

Table S7 - Time dependent AUC for qmAOM, AOM and M+BA.

Year	2.5	5	7.5	10
**qmAOM AUC (95% CI)**	0.88 (0.82-0.95)	0.83 (0.75-0.90)	0.86 (0.79-0.93)	0.81(0.69-0.92)
**AOM AUC (95% CI)**	0.77 (0.67-0.87)	0.76 (0.69-0.85)	0.75(0.65-0.85)	0.74(0.61-0.86)
**M+BA AUC (95% CI)**	0.78 (0.69-0.87)	0.75 (0.67-0.83)	0.75(0.66-0.84)	0.55(0.40-0.70)
**At Risk**	195	114	64	22
**Events**	21	38	51	61

AUC: Area under the curve; qmAOM: quantitative MRCP modified Amsterdam Oxford Model; CI: Confidence intervals; AOM: Amsterdam Oxford Model; M+BA: MRCP+ bilirubin and aspartate aminotransferase score.

Table S8 - Time dependent AUC for qmAOM and ANALI without contrast as a subset analysis of all patients with available ANALI score data

Year	1	2	3	4	5
**qmAOM AUC (95% CI)**	0.73 (0.51-0.95)	0.79 (0.62-0.95)	0.77 (0.63-0.91)	0.83 (0.70-0.95)	0.75 (0.58-0.92)
**ANALI_1 AUC (95% CI)**	0.51 (0.41-0.63)	0.66 (0.52-0.81)	0.77 (0.65-0.88)	0.80 (0.70-0.90)	0.75 (0.60-0.89)
**ANALI_2 AUC (95% CI)**	0.63 (0.46-0.81)	0.76 (0.63-0.90)	0.84 (0.74-0.93)	0.85 (0.76-0.93)	0.70 (0.55-0.87)
**At Risk**	104	97	89	67	21
**Events**	4	7	11	14	16

AUC: Area under the curve; qmAOM: quantitative MRCP modified Amsterdam Oxford Model; CI: Confidence intervals

ANALI_1: Combination of Oxford reviewer 1 and Monza/Bergamo reviewer

ANALI_2: Combination of Oxford reviewer 2 and Monza/Bergamo reviewer
