# Supplementary material for: Quantitative magnetic resonance cholangiopancreatography metrics improve prognostication in primary sclerosing cholangitis
Source: JHEP Rep. 2026 May 12;8(8):101892. doi: 10.1016/j.jhepr.2026.101892 (PMC13320491; doi:10.1016/j.jhepr.2026.101892)
Supplement: Multimedia component 2 [file mmc2.docx]

**JHEP Reports**

**CTAT methods**

Tables for a “Complete, Transparent, Accurate and Timely account” (CTAT) are now mandatory for all revised submissions. The aim is to enhance the reproducibility of methods.

- Refer to the CTAT in the main text as ‘Supplementary CTAT Table’
  1. **Software**

| **Software name** | **Manufacturer** | **Version** |
| --- | --- | --- |
| RStudio | Posit software, PBC | 2024.12.1- Build 563. |
| MRCP+ | Perspectum Ltd. | V2 |

- 1. **Please provide the details of the corresponding methods author for the manuscript:**

| Drs. T.E. Middelburg, MD  Department of Gastroenterology and Hepatology  Amsterdam UMC, location: VUmc  Room PK1 BR-118 I De Boelelaan 1118, 1081 HZ Amsterdam  E: [t.e.middelburg@amsterdamumc.nl](mailto:t.e.middelburg@amsterdamumc.nl)  T: +31650091357 |
| --- |
