# Supplementary material for: Quantitative magnetic resonance cholangiopancreatography metrics improve prognostication in primary sclerosing cholangitis
Source: JHEP Rep. 2026 May 12;8(8):101892. doi: 10.1016/j.jhepr.2026.101892 (PMC13320491; doi:10.1016/j.jhepr.2026.101892)
Supplement: Multimedia component 4 [file mmc4.pdf]

Research article

JHEP|Reports

Quantitative magnetic resonance cholangiopancreatography metrics improve prognostication in primary sclerosing cholangitis

Authors

Tim E. Middelburg, Laura Cristoferi, Willemijn Ponsioen, …, Jaap Stoker, Emma L. Culver, Cyriel Y. Ponsioen

Correspondence

t.e.middelburg@amsterdamumc.nl (T.E. Middelburg).

Graphical abstract

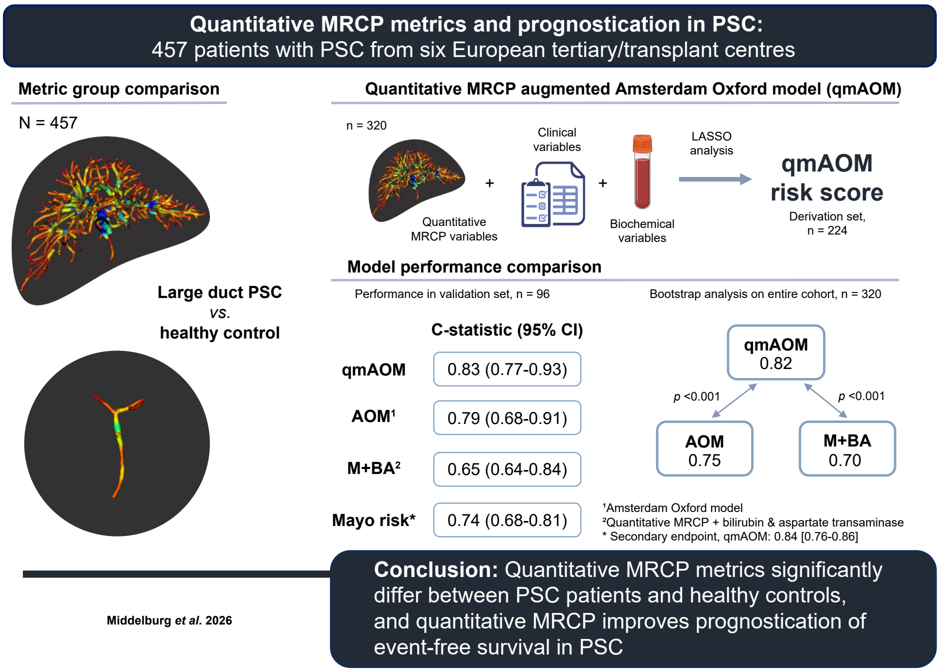

Highlights:

- First reference ranges established for quantitative MRCP metrics in PSC.
- Quantitative MRCP metrics differ markedly between patients with PSC and healthy controls.
- MRCP-derived biliary metrics add prognostic information to clinical variables.
- The qmAOM outperformed AOM and M+BA in predicting PSC-related events.
- Quantitative biliary features contribute to risk stratification in PSC.

Impact and implications:

This study establishes a reference range for quantitative MRCP metrics in primary sclerosing cholangitis and demonstrates their additive value predicting event-free survival. These findings support the incorporation of multidomain approaches in prognostic modelling and potential of quantitative imaging as surrogate endpoint in clinical trials.

https://doi.org/10.1016/j.jhepr.2026.101892

JHEP|Reports

Quantitative magnetic resonance cholangiopancreatography metrics improve prognostication in primary sclerosing cholangitis

Tim E. Middelburg[1,2,*,†], **Laura Cristoferi**[3,†], Willemijn Ponsioen[1,2], Maud Turkenburg[1,2], Carlos Ferreira[4], Tom Davis[4], Karin Horsthuis[5], Ynte S. de Boer[1,2], Adriaan J. van der Meer[6], Annemarie C. de Vries[6], Roy S. Dwarkasing[7], Johannes A. Bogaards[8], Sarah Al-Shakhshir[9,10], Palak Trivedi[9,10], Daphne D'Amato[3], Mauro Vigano[11], Eugenia V. Pesatori[11], Cesare Maino[12], Marco Carbone[3], Michael Pavlides[13,14], Jaap Stoker[2,5], Emma L. Culver[13,‡], Cyriel Y. Ponsioen[1,2,‡]

JHEP Reports **2026**. vol. 8 | 1–10

Check for updates

Background & Aims: Quantitative magnetic resonance cholangiopancreatography (MRCP) metrics showed prognostic potential in primary sclerosing cholangitis (PSC). This international multicentre study aimed to provide the first reference range for quantitative metrics in PSC and assess the additional role of quantitative metrics in prognostication.

Methods: Quantified MRCP (MRCP+) and clinical data were collected from six European tertiary referral and transplantation centres. A healthy cohort was available for comparison. Least Absolute Shrinkage and Selection Operator analysis was used to derive variables for a prognostic model in a derivation cohort, referred to as the *quantitative MRCP augmented Amsterdam–Oxford Model* (qmAOM). Composite endpoint included PSC-related mortality, transplantation, or hepatic decompensation, without hepatic decompensation as secondary endpoint. Performance was assessed in the validation cohort, expressed as C-statistic (95% CI), and compared with the Amsterdam–Oxford Model (AOM), Mayo Risk Score and MRCP+, bilirubin, and aspartate aminotransferase (M+BA) scores.

Results: In total, 457 participants with PSC were included for the reference range, which differed significantly in comparison with the healthy controls. Prognostic analysis included 320 participants. qmAOM included: age at PSC diagnosis, inflammatory bowel disease presence, serum alkaline phosphatase, aspartate aminotransferase, albumin, bilirubin, platelets, number of strictures, number of ducts with strictures or dilatations and proportion of 3–5-mm ducts. qmAOM, AOM, and M+BA demonstrated 0.83 (0.77–0.93), 0.79 (0.68–0.91), and 0.65 (0.64-0.84), respectively. In secondary endpoint analysis, qmAOM and Mayo Risk Score demonstrated 0.84 (0.76–0.86) and 0.74 (0.68–0.81), respectively. In bootstrap analysis using the entire prognostic cohort, qmAOM performed significantly better than AOM and M+BA (0.82; 0.75; 0.70, respectively; p <0.001).

Conclusions: This study provides the first reference range of quantitative metrics in PSC and indicates that quantitative biliary metrics improve discriminative performance in event-free survival in PSC.

© 2026 The Authors. Published by Elsevier B.V. on behalf of European Association for the Study of the Liver (EASL). This is an open access article under the CC BY license (http://creativecommons.org/licenses/by/4.0/).

Introduction

Primary sclerosing cholangitis (PSC) is a cholestatic liver disease, characterised by progressive inflammation and fibrosis of the biliary tree, leading to biliary stricturing and progressive liver disease. PSC follows a slow course, with a median time of 13–21 years from diagnosis to liver transplantation (LT) or death.[1] Furthermore, individuals with PSC are at high risk of developing cholangiocarcinoma and gallbladder carcinoma.[2] Currently, LT is the only curative option and there is no medical treatment that halts disease progression, while treatment development is hindered by the slow course to solid clinical endpoints. Therefore, the International PSC Study group (IPSCSG) declared the lack of validated surrogate endpoints for the natural history and progression of disease one of the biggest unmet needs in PSC.[3]

Several biochemical markers have been used to monitor disease activity, including serum alkaline phosphatase (ALP), bilirubin, and aminotransferase aspartate (AST). Risk scores based on biochemical marker such as the Mayo Risk Score, Amsterdam–Oxford Model (AOM), Primary Sclerosing Cholangitis Risk Estimate Tool (PRESTO) and UK-PSC score were developed to predict transplantation-free or decompensation-free survival in participants with PSC by using cholestatic and liver function related biomarkers.[4–7] However, their prognostic utility is limited by the fluctuating course of these markers, which reduces their long-term reliability. In parallel, radiological features such as magnetic resonance imaging (MRI), including magnetic resonance cholangiopancreatography (MRCP), are increasingly used in clinical practice. The DiStrict score and ANALI risk scores were developed to assess the extent of extra- and intrahepatic biliary alterations or parenchymal changes, thereby potentially capturing both disease severity and its complications.[8,9] Yet, radiological scores rely on semiquantitative assessments that remain prone to

* Corresponding author. Address: Department of Gastroenterology & Hepatology, Amsterdam UMC, De Boelelaan 1118, 1081 HZ Amsterdam, The Netherlands.
E-mail address: t.e.middelburg@amsterdamumc.nl (T.E. Middelburg).
† TEM and LC contributed equally as first authors.
‡ ELC and CYP contributed equally as senior authors.
https://doi.org/10.1016/j.jhepr.2026.101892

interobserver variability, hampering their reproducibility and standardisation. Evidence for the combination of domains is also scarce.[10] Given the heterogeneity of PSC, it seems appealing that new risk scores should incorporate robust information from multiple domains to achieve optimal prognostic performance.

Describing metrics of biliary anatomy, quantified by an MRCP post-processing software (MRCP+™, Perspectum Ltd, Oxford, UK), have been introduced as new potential reproducible and repeatable biomarkers devoid of interobserver variation.[11] Previous publications across different cohorts showed correlations with cholestatic laboratory biomarkers, imaging scores such as ANALI and the modified Amsterdam MRI score and its potential in predicting event-free, for example transplantation-free or decompensation-free survival in PSC. Total number of candidate strictures and the proportion of bile ducts with a 3–5-mm diameter are metrics that were repeatedly identified as prognostic metrics. Unfortunately, an extensive overview of metric ranges in PSC, and the comparison with healthy individuals, are lacking. Moreover, previous studies used different endpoints and previous studies lacked sufficient parameters and the statistical power to investigate the additional value of quantitative MRCP metrics to existing prognostic models.[10,12–16]

Therefore, the aim of this international collaborative study is to provide a reference range of quantitative metrics in PSC in comparison with values derived from healthy volunteers, assess the validity of previously identified metrics for the prediction of event-free survival in PSC, and assess the value of quantitative MRCP when incorporated into established risk score components.

Patients and methods

Study design

This study used existing cohorts from both tertiary care and transplantation centres across Europe that were updated to ensure maximal follow-up since acquisition of MRI.[13,15–17] Data were collected from July 2004 to March 2025 from six academic centres based in Italy (Monza, Bergamo), the UK (Birmingham, Oxford), and the Netherlands (Amsterdam, Rotterdam). Bergamo, Birmingham, and Rotterdam are transplant centres. The tertiary referral non-transplant centres were located in Monza, Oxford, and Amsterdam. The study was conducted in agreement with the ethical guidelines of the Declaration of Helsinki. Informed consent was given by participants in all centres for analysis of MRI data. All data was pseudonymised before analysis.

Selection of participants and MRI scans

All cohorts included patients with large-duct PSC, and some cohorts additionally included patients with small-duct PSC, provided a 3D-MRCP examination was available. PSC diagnosis was established in all participants according to definitions of the IPSCSG, EASL, UK-PSC, and AASLD guidelines.[18–21] Participants with features of autoimmune hepatitis (AIH) variant syndrome were included. Participants with LT or biliary surgery other than cholecystectomy, before the MRCP acquisition were excluded. Study designs and purposes varied across centres, differences in selection are described in Table S1. Data from Amsterdam/Rotterdam and Monza/Bergamo were previously collected for a prognostic evaluation purpose, whereas data from Birmingham and Oxford were collected for a prospective observational and technical purpose. For this study, the available data from all centres was reused and updated to maximise the follow-up per participant. The included MRCPs had centre-specific parameters, such as the anteroposterior coverage ranging from 66 to 240 mm. As liver-related measurements were not known, proportional coverage could not be provided. Further detailed information is shown in Table S2. A set of MRCP+ data of healthy volunteers, defined as no known liver or biliary disease, was provided and reused for comparison to aid differentiation of the observed absolute metric values in PSC from normal values. See Harisinghani *et al.*[22] for full details on the healthy volunteers.

MRCP+ analysis

Pseudonymised 3D-MRCP images were previously transferred to a portal hosted by Perspectum Ltd. for post-processing analysis by MRCP+™ by each centre. Quality control was performed by operators, trained in radiological assessment of the hepatobiliary region and software operation, by assessing breathing or motion artefacts, lack of biliary signal or over-projection of gastrointestinal fluids to exclude unreliable scans. The post-processing software identified tubular structures (ducts) exceeding the effective MRCP visibility threshold and generated a quantitative, signal-derived model of the entire biliary tree including the cystic duct, while excluding the pancreatic duct and gallbladder, as shown in Fig. 1. This model provides a signal-derived description of biliary morphology. For this study, MRCP+ quantification was performed on the entire biliary tree without anatomical sub-segmentation. MRCP + generates a large set of metrics; however, statistical analysis would be impeded by the number of metrics in comparison with the total number of events. Therefore, a selection of previously identified prognostic MRCP+ metrics was used to ensure reliable results. In total, five previously identified prognostic metrics by MRCP+ were explored: proportion of ducts with 3–5-mm diameter, number of strictures, total sum of length of strictures (mm), number of ducts with strictures or dilatations, and median duct diameter.[10–16] A stricture was defined as a local minimum in modelled duct widths, whose diameter is narrowed in comparison with adjacent ducts on either side of the stricture, by ≥1 mm in absolute terms and ≥30% in relative terms, while dilatation was defined as a local maximum with ≥30% relative and ≥1 mm absolute dilatation, in comparison with adjacent ducts on either side.[22]

Both complete loss of ductal signal, thereby causing separation of the ducts, and the absence of signal on either side of a narrowed or dilated duct (e.g. a distal prepapillar stricture), are not eligible to match these criteria and are not identified as strictures by the software. Therefore, the resulting and related metrics may not represent exact anatomical counts of strictures or ducts in all cases. Quantification results were not compared with original images by expert radiologists in this study.

Clinical data collection

The following demographic and clinical data were collected: Age at inclusion, sex at birth, small- or large-duct PSC and

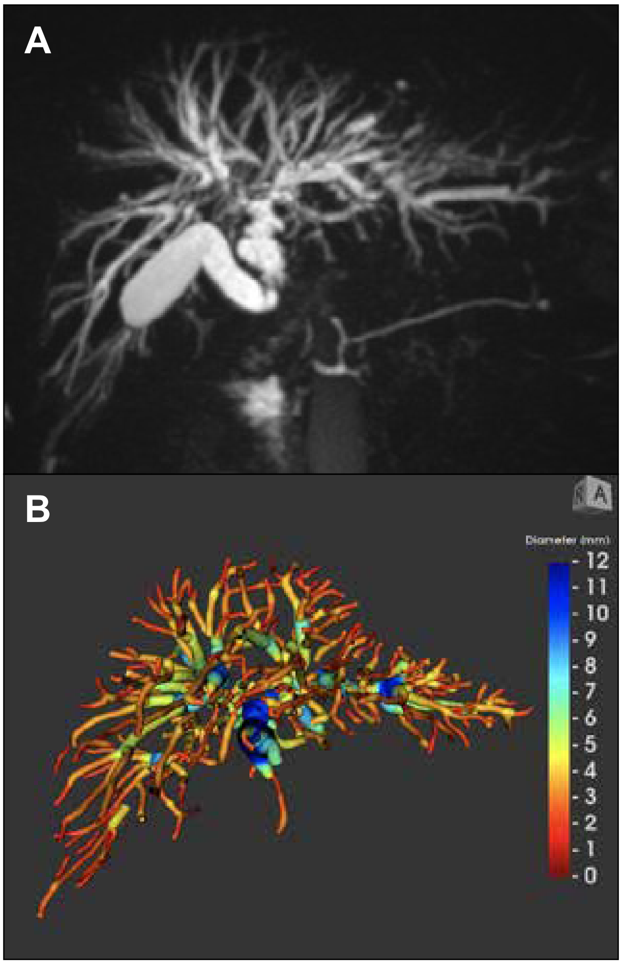

Fig. 1. Conversion of MRCP to a MRCP+ model in a 51-year-old PSC patient, 20 years after diagnosis of PSC. (A) Maximum intensity projection of a 3D-MRCP and (B) the corresponding MRCP+ model (including the colour coding by duct diameter). The gallbladder and pancreatic duct were excluded from post-processing. In this MRCP+ model, the number of visible ducts were 244, 81 ducts with a 3–5-mm diameter, 34% proportion of ducts with a 3–5-mm diameter. Number of strictures was 31, sum of stricture length was 278 mm, number of dilated ducts was 74, total length of dilated ducts was 476 mm, and number of strictures or dilated ducts was 63. MRCP, magnetic resonance cholangiopancreatography; PSC, primary sclerosing cholangitis.

date of PSC diagnosis, presence and type of inflammatory bowel disease (IBD), closest laboratory evaluation to MRCP date (±3 months), clinical score (AOM and Mayo Risk Score), composite score of quantitative MRCP with serum bilirubin and AST (M+BA), and radiological scores (ANALI). AOM, M+BA, and Mayo Risk Scores were treated as continuous variables, whereas the ANALI score was treated as an ordinal variable ranging from 0 to 5. ANALI scores from both Oxford and Monza/Bergamo were available. In the Oxford cohort, two expert radiologists performed ANALI scoring independently. In the Monza/Bergamo cohort, a single expert-derived ANALI score was available. For the Mayo Risk Score, reported variceal bleeding prior to MRI acquisition was used. For endpoint analysis, data regarding death, transplantation and hepatic decompensation were collected. Hepatic decompensation was considered as absence or presence of ascites on ultrasound or MRI, hepatic encephalopathy, or variceal bleeding. Ascites was recorded as present or absent based on radiologist report and was not categorised by extent or quantified.

Statistical analysis

Categorical data are displayed as numbers (percentages) and continuous data as mean (±SD) or median (IQRs, Q1–Q3). A composite primary endpoint was used, consisting of hepatic decompensation, LT or PSC-related death, excluding death from colorectal carcinoma (CRC). PSC-related death included: death by end-stage liver disease and its complications, cholangitis-related events, death by cholangio-, gallbladder- or hepatocellular carcinoma. CRC was excluded as it is not deemed to be reflected by cholestatic biomarkers. Secondary endpoints were: (1) composite of LT or PSC-related death without CRC; (2) composite of death by all causes or LT. Participants that did not experience an event were censored at the last moment of follow-up.

To explore quantitative metric differences between type of PSC and healthy volunteers, all participants with PSC from all centres were used. The Kruskal–Wallis test was used for comparison between the groups and pairwise Wilcoxon rank-sum tests for *post-hoc* pairwise comparison. *p* values were two-sided, and $p < 0.05$ was considered statistically significant. To assess the prognostic use of quantitative metrics, PSC participants with at least 1 year of event-free follow-up and available laboratory data were used. Consistent with previous literature, IBD, including ulcerative colitis (UC), Crohn's disease (CD), and IBD-unclassified (IBD-U), was dichotomously categorised as diagnosis of IBD (CD, UC, and IBD-U) *vs.* no IBD. This approach reduces subtype misclassification and avoids unstable effect estimates caused by low numbers in CD and IBD-U subgroups, thereby improving robustness of variable selection in modelling.[5]

The following variables were considered for model development: five pre-selected prognostic MRCP+ metrics, clinical features (age at diagnosis; age at MRCP; time from diagnosis of PSC to MRCP acquisition; presence of UC; and type of PSC), centre type of inclusion (tertiary care or transplantation centre), and laboratory parameters (ALP; bilirubin; AST; ALT; albumin and platelets). Laboratory values were rationed to the upper or lower limit of normal and normalised via log-transformation.

To identify robust and stable prognostic predictors, variable selection was performed using Least Absolute Shrinkage and Selection Operator (LASSO) within a bootstrap framework applied to the entire cohort with 500 iterations.[23,24] In each bootstrap iteration, predictors were standardised, and a LASSO penalty was applied to shrink regression coefficients toward zero; depending on the penalty strength (λ), some coefficients were reduced to exactly zero, thereby excluding those predictors from the model. The optimal λ was selected by cross-validation to limit overfitting. Predictors with non-zero coefficients were retained for that iteration. Predictor stability was quantified as the proportion of bootstrap resamples in which each predictor was selected (selection frequency), and predictors with selection frequency >50% were retained. To evaluate model performance and generalisability, the cohort was randomly split into derivation and validation sets in a 70:30

ratio. The predictors retained from the bootstrap-LASSO procedure were implemented in a final LASSO model fitted in the derivation set to estimate penalised coefficients and construct a multivariable prognostic model, referred to as the quantitative MRCP modified AOM (qmAOM), calculated as a weighted linear combination of the selected predictors, from which an individual risk score was derived.

To assess discriminative performance, Harrell's C-statistic was determined, and a refitted model excluding quantitative metrics was derived via LASSO in the derivation set to evaluate the additional performance of quantitative metrics. To assess stratification, the optimal risk score cut-off was determined via maximally selected rank statistics.[25] This method ensures a maximised separation of outcome groups based on event and survival time. Based on this cut-off, low and high-risk groups were created after which between-group hazard ratios (HRs) with CIs were assessed by Cox regression analysis. Risk group survival differences were visualised with Kaplan–Meier survival curves. Accuracy over time was assessed with time-dependent C-statistics and depicted as area under the receiver operator curve (ROC). To assess model calibration, mean model-predicted survival probability were compared with the observed Kaplan–Meier survival curve in the validation set. The Brier score (equivalent to the mean squared error when applied to predicted probabilities) was determined to evaluate predictive accuracy, for which a Brier score <0.10 is deemed good and 0.11–0.15 moderate.[26]

For the secondary endpoints, participants with at least 1 year of transplantation-free survival were included and prognostic performance of the qmAOM, as derived from the primary endpoint, was assessed without splitting the cohort in advance. Predictive performance (C-statistic) of the original AOM, M+BA, and Mayo Risk Score was assessed in identical cohorts as the qmAOM to ensure a fair descriptive comparison of model discrimination. The Mayo Risk Score was not compared for the primary endpoint because this endpoint contains the variceal bleeding component used to calculate the Mayo Risk Score. Given the derivation-validation design, the un-nested model structures, and the presence of right-censoring, comparison initially focused on the direction and magnitude of differences between C-statistics, recognising the limited power of formal significance testing. However, to explore the discriminative potential, a bootstrap-based (n = 500) analysis was performed in the entire cohort to compare Harrell's C-statistic of the risk scores.

As exploratory analysis, the prognostic performance of the ANALI score without gadolinium, scored in the Oxford and Monza/Bergamo cohorts, was assessed using the same C-statistic analysis as described previously and the correlation with the qmAOM and its individual quantitative metrics was assessed via Spearman's rank correlation. As no formal consensus score was available for Oxford, both readers were analysed separately and each Oxford reader's scores were pooled with the Monza/Bergamo cohort, resulting in two parallel combined analyses.

R version 2024.12.1 (R Foundation for Statistical Computing, Vienna, Austria) was used for statistical analysis and key packages included *gtsummary* and *Hmisc* for descriptive statistics, *glmnet* for LASSO regression, *survival* for Cox regression and Kaplan–Meier analysis, *survminer* and *ggplot2* for visualisation, *pec* for calibration analysis and *pROC* and *timeROC* for ROC analysis. The CTAT is included in the supplementary CTAT table.

Results

Demographics

A total of 457 participants with PSC were included in this multicentre study, as shown in Fig. 2. Most participants had large-duct PSC (93.1%) as shown in Table 1, and a median follow-up of 11.4 years after diagnosis of PSC and 5.2 years after the date of the MRCP. There were no significant differences found between centre types, except for serum albumin, as shown in Table S3. The study included a total of 59 healthy controls, with similar demographics (Table 1).

Comparison with healthy controls

Most of the metrics demonstrated a significant difference between large-duct PSC *vs.* small-duct PSC and healthy controls, such as number of visible ducts (74 *vs.* 27 *vs.* 18, respectively, p <0.001) and total length of strictures (68.4 mm *vs.* 19.3 mm *vs.* 12.9, respectively, p <0.05) in mm, except for the proportion of ducts with a median 3–5-mm diameter as shown in Table 2. The visual difference between a healthy

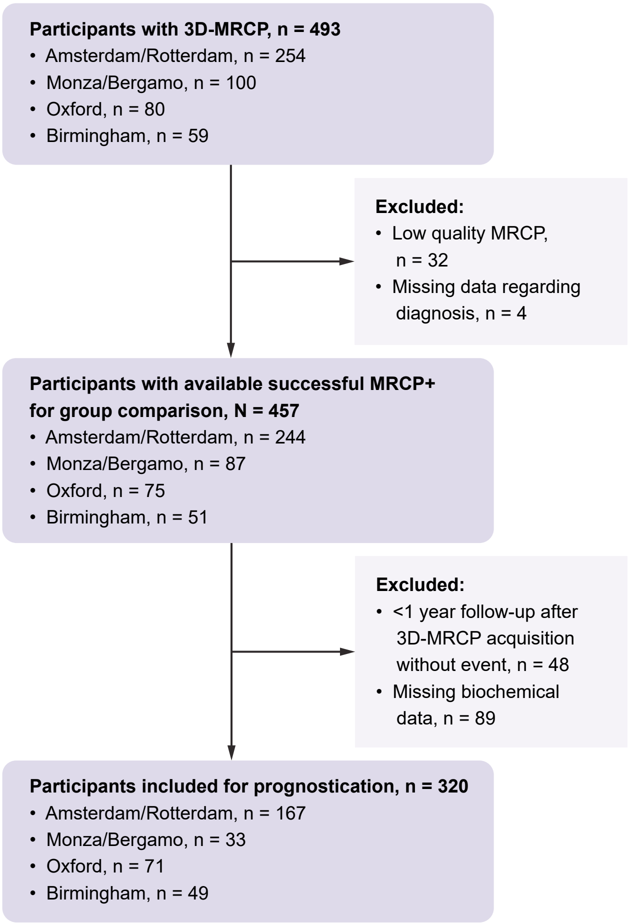

Fig. 2. Flowchart of MRCP inclusion. MRCP, magnetic resonance cholangiopancreatography.

Table 1. Patient demographics and MRCP+ characteristics per PSC disease type and healthy controls.

	Large-duct PSC	Small-duct PSC	Healthy controls
	n = 427	n = 30	n = 59
Sex, male	275 (64.4)	19 (63.3)	39 (66.1)
Age at PSC diagnosis (years)	36.4 (25.7, 47.4)	32.3 (20.3, 51.5)	—
Centre type			
Tertiary	203 (47.5)	22 (73.3)	—
Transplantation	224 (52.5)	8 (26.7)	—
Type of IBD	180 (42.2)	16 (53.3)	—
None			
UC	215 (50.4)	13 (43.3)	—
CD	25 (5.9)	0 (0)	—
IBD-U	7 (1.6)	1 (3.3)	—
Age at MRCP+ (yr)	46.0 (31.5, 55.0)	39.0 (28.5, 53.0)	43.0 (33.0–54.0)
PSC diagnosis to MRCP+ (yr)	5.7 (1.2, 10.9)	2.8 (0.1, 8.7)	—
MRCP+ to event or censoring (yr)	4.8 (3.3, 7.9)	7.8 (4.7, 9.3)	—
PSC diagnosis to last follow-up (yr)	11.4 (7.1, 16.7)	10.8 (7.0, 16.4)	—
ALP (U/L), n = 429	203 (112, 348)	185 (102, 306)	—
Bilirubin (µmol/L), n = 418	14 (10, 28)	9 (7, 15)	—
Albumin (g/L), n = 386	41 (38, 45)	43 (41, 45)	—
AST (U/L), n = 430	50 (30, 87)	41 (29, 68)	—
Platelets ($\times 10^9$/L), n = 414	250 (190, 315)	279 (243, 338)	—
AOM risk score, n = 343/21	1.6 (1.3, 2.1)	1.1 (0.7, 1.3)	—
AOM low risk (<2.00), n = 343/21	236 (68.9)	21 (100.0)	—
Mayo Risk Score, n = 322/24	-0.12 (-0.75, 0.54)	-0.64 (-1.06, -0.42)	—
Mayo low risk (<0), n = 322/24	182 (56.5)	21 (87.5)	—
Events - primary endpoint*			
Hepatic decompensation	80 (18.4)	4 (13.3)	—
Transplantation	32 (7.0)	0 (0)	—
PSC-related death excl. CRC	39 (8.5)	0 (0)	—
Events - secondary endpoint			
PSCR-death or LT incl. CRC	123 (26.9)	4 (13.3)	—
PSCR-death or LT excl. CRC	122 (26.7)	4 (13.3)	—

Reference ranges: ALP ≤120 U/L; bilirubin ≤18 µmol/L; albumin ≥35 g/L; AST ≤35 U/L; platelets ≥150 × 10^9/L. Continuous numbers are displayed as n (%) or median (Q1, Q3). *The composite primary endpoint shows which event occurred first in follow-up after MRCP+. ALP, alkaline phosphatase; AOM, Amsterdam–Oxford Model; AST, aspartate aminotransferase; CD, Crohn's Disease; CRC, colorectal carcinoma; IBD, inflammatory bowel disease; IBD-U, unclassified IBD; LT, liver transplantation; MRCP, magnetic resonance cholangiopancreatography; n, number of participants; PSC, primary sclerosing cholangitis; UC; ulcerative colitis.

control *vs.* small-duct PSC *vs.* large-duct PSC with a similar proportion of ducts with a 3–5-mm diameter is demonstrated in Fig. 3.

Composing qmAOM

For prognostic analysis, 48 participants were excluded because they had <1 year of follow-up before meeting the primary endpoint and 89 were excluded because of missing biochemical data. A total of 320 participants were included, with a median follow-up of 5.1 years after acquisition of the MRCP and 88 participants met the primary endpoint. Pre-selection identified 11 variables exceeding 50% selection in bootstrap analysis: Serum albumin, total bilirubin, platelets, ALP, AST, age at PSC diagnosis, IBD, centre type, proportion of ducts with a 3–5-mm duct diameter, total number of strictures and number of ducts with strictures or dilatations, as shown in Table S4.

The cohort was split into a derivation and validation set and no significant differences were observed between the two sets, as shown in Table S5. The following variables were included in the final prognostic model, selected by LASSO: age at PSC diagnosis, presence of IBD, log of ALP × upper limit of normal (ULN), log of total bilirubin × ULN, log of albumin × lower limit of normal (LLN), log of AST × ULN, log of platelets × LLN, proportion of ducts with a 3–5-mm duct diameter, total number of strictures, number of ducts with strictures or dilatations.

Table 2. MRCP+ characteristics per disease type and healthy controls.

	Large-duct PSC	Small-duct PSC	Healthy
	n = 427	n = 30	n = 59
Number of visible ducts	74 (42, 131)*	27 (18, 38)†	18 (13, 24)
Total length of visible ducts (cm)	38 (27, 54)	52 (37, 76)	141 (79, 257)
Number of ducts with 3–5 mm diameter	12 (7, 27)*	6 (3, 7)‡	4 (3, 5]
Proportion of ducts with 3–5 mm diameter (%)	21.2 (13.4, 29.5)	21.8 (15.4, 29.0)	22.7 (15.4, 29.5)
Number of strictures	9 (5, 17)*	3 (2, 4)‡	2 (1, 3)
Total length of strictures (mm)	68.4 (35.4, 126.3)*	19.3 (12.0, 34.5)	12.9 (6.1, 28.7)
Number of dilated ducts	17 (9, 32)*	5 (3, 7)†	2 (2, 4)
Total length of dilated ducts (mm)	108.4 (62.0, 207.3)*	35.1 (23.7, 46.8)‡	26.9 (15.7, 39.0)
Number of strictures or dilated ducts	17 (9, 30)*	5 (4, 7)†	3 (2, 4)

All values are displayed as median (Q1, Q3). **p* <0.001 *vs.* all groups; †*p* <0.001 *vs.* healthy controls; ‡*p* <0.05 *vs.* healthy. Group differences were assessed with Kruskal-Wallis test and Wilcoxon rank-sum test.

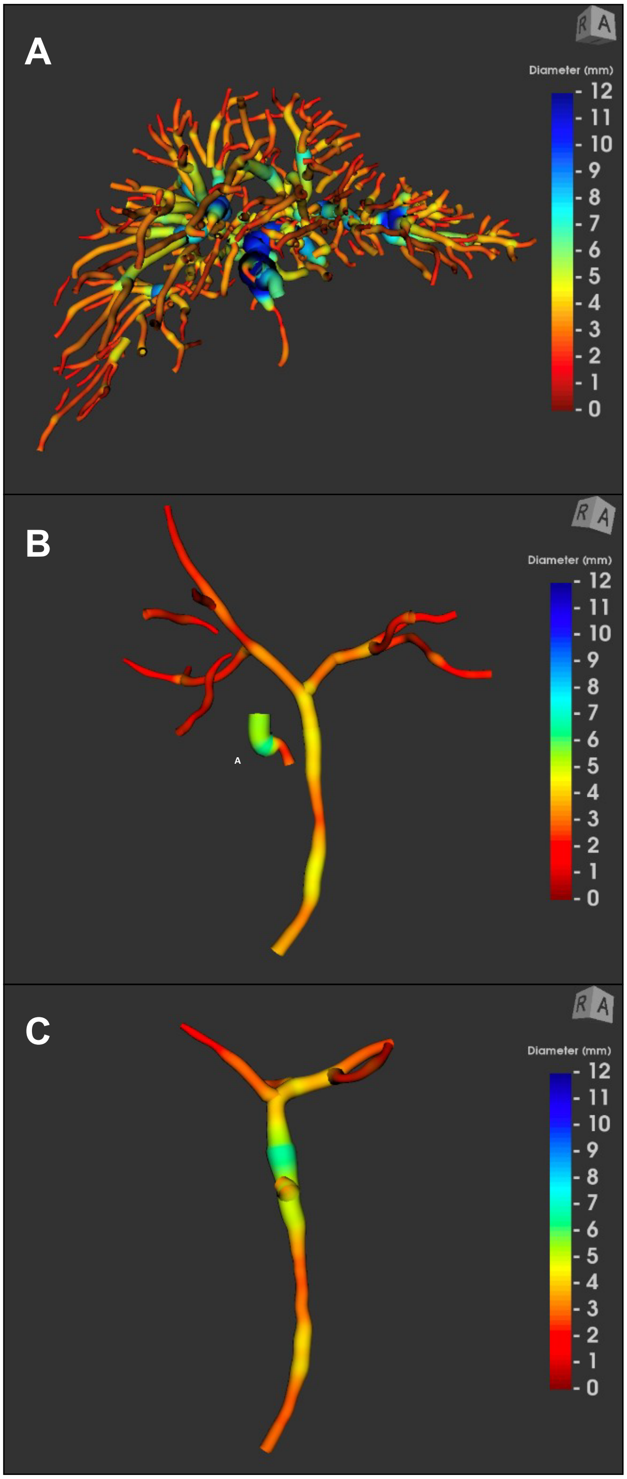

Fig. 3. Visual difference between MRCP+ with similar proportion of ducts with median 3–5-mm diameter. (A) Visualises the MRCP+ acquisition of a large-duct PSC patient with a 34% (83/244 ducts with median 3–5-mm diameter) proportion of median 3–5-mm diameter ducts while (B) shows a small-duct patient with a 37% (10/27 ducts) proportion and (C) a healthy control with a 37.5% (three of eight ducts) proportion of 3–5-mm diameter ducts. This figure emphasises the increased load of intrahepatic ducts that have crossed the 1 mm MRI resolution threshold in patients with PSC. MRCP, magnetic resonance cholangiopancreatography; MRI, magnetic resonance imaging; PSC, primary sclerosing cholangitis.

These variables were combined to compose the risk formula *qmAOM*, including their penalised regression weights that represent variable contribution, as shown below:

Formula 1 - Formula of the qmAOM risk score

qmAOM score =
*0.008 * Age at PSC diagnosis + 0.379 * IBD + 0.725 * Serum ALP + 0.794 * Serum total bilirubin – 3.070 * Serum albumin – 0.899 * Platelets + 0.403 * Serum AST + 0.019 * Proportion of 3-5* mm *ducts (%) + 0.011 * Total number of strictures + 0.005 * Number of ducts with strictures or dilatations*

Age at PSC diagnosis in years; IBD present = 1, absent = 0; AST, ALP and total bilirubin are expressed in x the ULN and transformed to log-scale; Albumin and platelets are expressed as x the LLN and transformed to log-scale. Presented coefficients are penalised regression weights from the LASSO model; they represent variable contribution rather than hazard ratios with confidence intervals.

The median qmAOM score in the derivation set was 0.86 (Q1, Q3: 0.43, 1.41) and the score ranged from -0.79 to 3.83.

Primary endpoint performance

For the primary endpoint (composite of hepatic decompensation, transplantation, or PSC-related death), the qmAOM score performance analysis showed a C-statistic of 0.82 (95% CI: 0.74–0.86) in the derivation set and 0.83 (95% CI: 0.77–0.93) in the validation set. By comparison, a similar model without the selected MRCP+ variables had a maximum of 0.75 (95% CI: 0.64–0.86) in the validation set, while a model including only MRCP+ metrics would not exceed a C-statistic of 0.70 (data not shown). Excluding SD-PSC for sensitivity resulted in C-statistics of 0.82 (95% CI: 0.74–0.87) and 0.82 (95% CI: 0.76–0.93) in the derivation and validation set, respectively.

The optimal threshold between low and high risk was identified at 1.29 and the HR between high and low-risk groups was 7.67 (95% CI: 4.57, 12.9) and 8.4 (95% CI: 3.31, 21.3) in the derivation and validation sets, respectively, as shown by the Kaplan–Meier curve and risk table in Fig. 4. The cut-off had a sensitivity and specificity of 61% and 85%, respectively. Long-term accuracy was stable at an AUC of 84% at year 10 and an overall AUC of 84%. All other timepoints from 2.5 and 10 years had AUCs >80% as shown in Fig. S1.

Calibration analysis of the qmAOM demonstrated slight overestimation of event-free survival over time in the validation set, as shown in Fig. S2 and Table S6. The time-dependent Brier score performed satisfactorily, being 0.05 (good accuracy) at year 2 and 0.14 (moderate accuracy) at year 10, as shown in Table S6.

Secondary endpoint performance

Including 331 PSC participants when filtered for at least 1 year of PSC-related transplant-free survival, the qmAOM showed a C-statistic of 0.84 (95% CI: 0.76–0.86) for the composite endpoint of LT or PSC-related death without CRC. For death from all causes or LT, the qmAOM demonstrated a C-statistic of 0.79 (95% CI: 0.73–0.84).

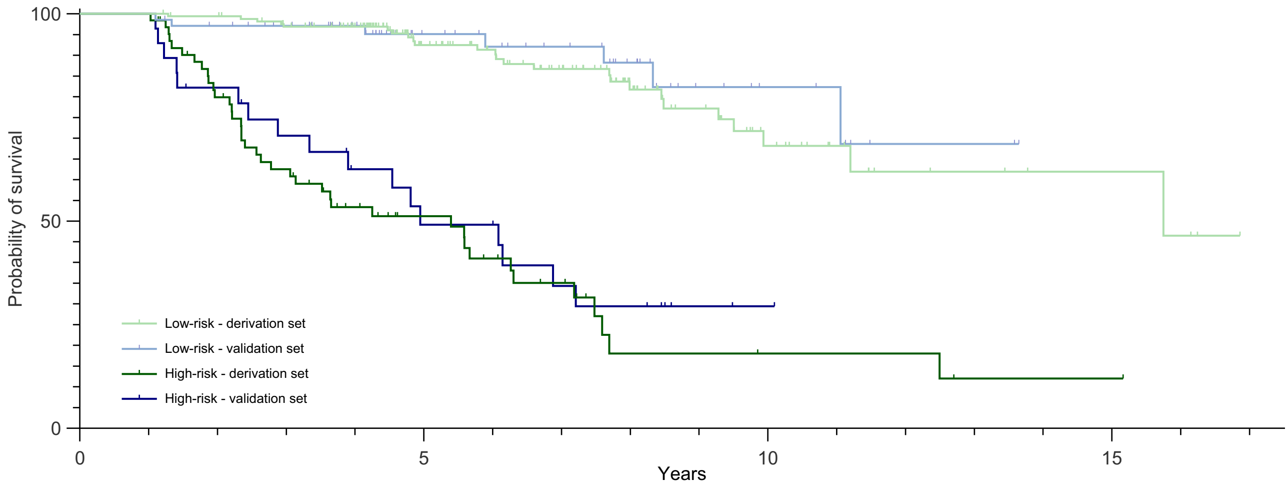

Risk group		0 years	4 years	8 years	12 years	16 years
Derivation: Low	At risk	162	139	44	7	3
	Censored	0	19	100	131	134
	Event	0	5	18	24	25
	Event ratio	0%	3.1%	11.7%	14.8%	15.4%
Validation: Low	At risk	68	51	19	2	0
	Censored	0	15	44	59	61
	Event	0	2	5	7	7
	Event ratio	0%	2.9%	7.3%	10.3%	10.3%
Derivation: High	At risk	62	26	4	3	0
	Censored	0	9	20	21	23
	Event	0	27	38	38	39
	Event ratio	0%	43.5%	61.3%	61.3%	62.9%
Validation: High	At risk	28	14	6	0	0
	Censored	0	4	5	11	11
	Event	0	10	17	17	17
	Event ratio	0%	35.7%	60.7%	60.7%	60.7%

Fig. 4. Kaplan–Meier curve of risk groups in both derivation and validation sets. Based on the 1.29 cut-off, low-risk and high-risk were created in both sets. Number of participants per group and set are shown in the risk table.

Performance comparison

Original AOM, M+BA, and Mayo Risk Score

Although both AOM and M+BA were not validated on the selected primary composite endpoint in this study, their performance was assessed. The AOM showed a performance of 0.74 (95% CI: 0.64–0.79) and 0.79 (95% CI: 0.68–0.91) in the derivation and validation sets, respectively. The M+BA score showed a C-statistic of 0.72 (95% CI: 0.65–0.78) in the derivation set and 0.65 (95% CI: 0.64–0.84) in the validation set.

Using the composite secondary endpoint of LT or PSC-related death without CRC for the original AOM, M+BA, and Mayo Risk Score, these scores demonstrated lower C-statistics of 0.73 (95% CI: 0.64–0.77, p <0.001), 0.72 (95% CI: 0.67-0.78, p <0.001), and 0.76 (95% CI: 0.68–0.81), respectively. For death from all causes or LT, the original AOM, M+BA, and Mayo Risk scores demonstrated lower C-statistics of 0.71 (95% CI: 0.63–0.76), 0.67 (95% CI: 0.63–0.75), and 0.74 (95% CI: 0.68–0.81), respectively.

The long-term accuracy of all scores was assessed over a 10-year period and qmAOM score showed a stable AUC over 10 years as shown in Table S7. Overall, the qmAOM demonstrated the highest AUC at all timepoints.

Bootstrap-based analysis of the entire cohort showed a higher C-statistic for qmAOM in comparison with the original AOM (0.82 *vs.* 0.75; p <0.001, respectively) and M+BA (0.82 *vs.* 0.70; p <0.001).

ANALI score without gadolinium

In total, 115 participants had a total ANALI score without gadolinium available, and 42 participants had an ANALI score with gadolinium available. Median ANALI score without gadolinium was between 1 (Q1–Q3: 0–3) and 2 (Q1–Q3: 0–3).

In this subset, qmAOM demonstrated a C-statistic of 0.78 (95% CI: 0.68–0.91), whereas the original AOM showed a C-statistic of 0.73 (95% CI: 0.62–0.86). Combining the Monza/Bergamo scores with scores of Oxford reviewer 1, the C-statistic was 0.73 (95% CI: 0.64–0.84), while use of Oxford reviewer 2 resulted in a C-statistic of 0.78 (95% CI: 0.67–0.86). In time-dependent analyses, qmAOM showed numerically

higher C-statistics up to 2 years, with alternating discrimination between ANALI and qmAOM thereafter as shown in Table S8.

In risk group stratification, the qmAOM demonstrated a HR of 4.35 (95% CI: 1.43–13.23; p = 0.009) between the low-*vs.* high-risk group, as shown in Fig. S3. Both combinations of ANALI without gadolinium from Oxford reviewer 1 and 2 with Monza/Bergamo showed significant risk group stratification (HR 2.92 [95% CI: 1.14–7.46] and HR 4.09 [95% CI: 1.57–10.64], respectively, as shown in Figs. S4 and S5).

Moderate correlation was found between the qmAOM score and total ANALI score of both combinations 1 and 2 (0.49 and 0.50, respectively). Moderate correlations were found in both combined assessments of intrahepatic dilatation scored by ANALI and number of ducts with a stricture or dilatation (0.65 and 0.59) and total number of strictures (0.59 and 0.53), as shown in Fig. S6.

Discussion

This international multicentre collaboration is the largest study on quantitative MRCP+ metrics and the first to provide an extensive overview of quantitative metric range in participants with PSC *vs.* healthy volunteers. Furthermore, this study confirms the accurate prognostic value of several quantitative MRCP metrics and establishes the additional value of quantitative MRCP metrics in prediction of long-term event-free survival in PSC, to produce a novel qmAOM risk score, even outperforming currently used risk scores for PSC.

The number of strictures, proportion of 3–5-mm ducts and number of ducts with stricture or dilatation were confirmed as aiding prognostication in PSC. Combined with the clinical domain (age at PSC diagnosis and presence of IBD) and laboratory domain (ALP, AST, albumin, total bilirubin, and platelets), this resulted in a risk score that demonstrated strong and stable performance over time with C-statistics above 0.80 in both derivation and validation sets. Furthermore, significant stratification performance was demonstrated in both sets, although a wider HR was observed in the validation set, likely caused by the smaller number of events. The qmAOM demonstrated mild overestimation of event-free survival in our study, however, the Brier score suggests excellent to good accuracy up to 4 years and moderate accuracy from year 4 to 10. This decrease in accuracy is likely caused by the decrease in available data with longer follow-up time and possibly by the heterogenous progression in PSC, indicating the need for risk re-evaluation with updated measurements over time. In comparison to the AOM and M+BA, the qmAOM outperformed both in terms of C-statistic. This was supported by bootstrapping, showing a significant difference between performance in the entire cohort. As expected in external validation, the M+BA score performed worse in comparison to the performance (C-statistic of 0.86) described in the original publication, most likely caused by less advanced disease stages and longer follow-up in our cohort.[10] Satisfactory performance was also demonstrated when using the secondary endpoint, PSC-related death or LT, underlining its robustness in both composite endpoints including hepatic decompensation, as in definite endpoints such as transplantation and death. For these endpoints, the qmAOM also showed numerical higher C-statistic than the Mayo Risk Score. This similar performance is most likely explained by the natural cascade of PSC progression, as hepatic decompensation precedes transplantation or death, and it is likely that the same participants encountered both the primary and secondary endpoint. Therefore, it was not unexpected that most of the primary endpoint consisted of hepatic decompensation.

The identified prognostic metrics were in line with previous studies.[10,12,13,16] Strictures are a common component of several risk scores by both MRCP and ERCP, and the rationale of including these variables in the qmAOM is in line with the nature of PSC and has apparent face validity. In contrast to the original AOM, our model incorporates these stricture-related metrics instead of PSC subtype. Given the significant differences observed between large- and small-duct PSC, these quantitative metrics likely capture a more nuanced, continuous reflection of disease severity rather than a dichotomous classification that assumes uniform risk among all large-duct participants. The inclusion of IBD presence in the qmAOM score is in line with described worse survival in patients with PSC-IBD when compared with patients with PSC-only. Furthermore, this clustering overcomes misclassification of IBD subtype, as subtypes are not infrequently difficult to distinguish, especially in patients with PSC.

Assessment of metric ranges showed significant differences between healthy controls, small-duct PSC, and large-duct PSC. However, no significant difference between groups was observed for the proportion of ducts with a 3–5-mm diameter, while this metric was repeatedly identified as a prognostic MRCP+ metric. Firstly, it is important to recognise that prognostically relevant features do not necessarily need to correlate with potential diagnostic features.[27] Second, this phenomenon is most likely caused by comparing different orders of ducts. As illustrated in Fig. 3, this proportion concerns central ducts in healthy controls and small-duct PSC participants, while in large-duct PSC, progressive obstruction with upstream dilatation allows visualisation of peripherally dilated ducts at MRCP+. This also supported by the differences in absolute number of ducts with a 3–5-mm median diameter. Although this metric could be inflated in presence of signal voids, it showed a similar increased trend as the sum of duct length that is independent of inflation by voids (data not shown). As this study includes PSC participants with widespread disease stages, the observed overlapping proportions can best be interpreted as consequence of group heterogeneity rather than a true pathophysiological similarity between the groups.

A strength of using MRCP+ metrics is that these are devoid of interobserver variability, in contrast to current radiological scores, because the radiological metrics are assessed by an independent algorithm. Consequently, the qmAOM combining several domains including clinical, biochemical, and radiological parameters which can all be assessed objectively, has the same advantage. The numerically higher C-statistic found for the qmAOM compared with the ANALI score without gadolinium demonstrated at least equal performance, without the potential limitation of subjectivity. This limitation was illustrated by differences in C-statistic between two reviewers from one centre. Another strength are the described high rates of technical success. Ranging between 87% and 95%, this supports the potential widespread availability, especially since the current MRCP+ algorithm is aligned with currently used protocols in clinical care.[13,16,17] Perspectum Ltd. states that current failure rates are below 5%, with the failures being related to imaging-quality issues. Therefore, these results call

for application in clinical trials for evaluation of MRCP+ metrics and/or qmAOM as surrogate endpoint.

Several limitations should be acknowledged. The retrospective nature of some of the cohorts may have introduced selection bias in the dataset. However, based on the prospective approach of both UK cohorts, combined with the nationwide data from the Netherlands, the cohort provided heterogeneity and an opportunity to assess disease severity across the entire disease spectrum. In support of reproducibility, the independent re-identification of the quantitative metrics by LASSO were concurrent with previous studies that were not part of this study. As for metric-specific limitations, the total number of strictures might be affected in opposite directions, depending on the extent of ductal stricturing. Local calibre variations might fit the stricture criteria, thereby possibly overestimating stricture count in individuals, as seen in the healthy control and small-duct PSC group. This might lower the specificity of stricture-related metrics. Strictures causing complete loss of signal or absence of adjacent reference ducts however, do not fit stricture criteria. This possibly causes underestimation of stricture-related counts and consecutively, identifying an increased in ducts because of ductal discontinuation. Despite these opposing effects, total number of strictures and stricture-related metrics were repeatedly identified as prognostic in independent cohorts, indicating that these metrics capture a prognostic signal of ductal disease burden rather than exact counts. Further optimisation of accurate stricture detection, particularly near the visibility threshold, remains warranted and is ongoing. This is expected to further augment the already substantially improved C-statistics of this model. Methodologically, the limited number of events hampered formal performance comparison between currently used risk scores such as the AOM. However, exploratory bootstrap analysis showed significantly higher C-statistic for qmAOM, although performance bias should be acknowledged as participants that were used in deriving the qmAOM score were also included in the bootstrap analysis. Unfortunately, ascites is not well defined and could be misinterpreted for normal physical levels of intraperitoneal fluids, for example in women during the menstrual cycle. However, experienced radiologists are able to distinguish ascites from physical levels, and we therefore deem it unlikely that this might have caused an overestimation of ascites-related primary endpoints. The limited amount of available ANALI with gadolinium contrast and the novel DiStrict score data precluded a robust comparison with the qmAOM. For the exploratory comparison with ANALI without gadolinium contrast, performance of the qmAOM might be biased as participants from the derivation set were reused, while fluctuations in AUC of the ANALI score without gadolinium are most likely the result of decreasing numbers at risk rather than true discrimination. This study focused only on quantitative biliary assessment. Therefore, the role of parenchymal assessment remains unclear, requiring future, preferably prospective studies. Ideally, these future studies would also include qualitative biliary and parenchymal assessment by radiologists to perform a direct quantitative *vs.* qualitative comparison. Currently, MRCP+ technology is valued at $950.50 in a hospital setting in the US.[28] In the UK a price point of up to £500 is deemed as being of value to the National Health System (NHS), indicating the necessity for additional prospective cost-benefit analysis of using quantification technology.[29]

In conclusion, this study provides an extensive reference range of quantitative MRCP metrics for individuals with PSC, confirms the prognostic value of several quantitative metrics, and confirms their use in improving prognostication. The qmAOM is shown to be a risk score with strong prognostic performance that outperforms other current risk scores.

Affiliations

[1]*Department of Gastroenterology & Hepatology, Amsterdam UMC, Amsterdam, The Netherlands;* [2]*Amsterdam Gastroenterology, Endocrinology & Metabolism, Amsterdam, The Netherlands;* [3]*Division of Gastroenterology, Centre for Autoimmune Liver Diseases, European Reference Network on Hepatological Diseases (ERN RARE-LIVER), Fondazione IRCCS San Gerardo dei Tintori, Monza, Italy;* [4]*Perspectum Ltd., Oxford, UK;* [5]*Department of Radiology and Nuclear Medicine, Amsterdam UMC, University of Amsterdam, Amsterdam, The Netherlands;* [6]*Department of Gastroenterology & Hepatology, Erasmus MC, Rotterdam, The Netherlands;* [7]*Department of Radiology and Nuclear Medicine, Erasmus MC, Rotterdam, The Netherlands;* [8]*Department of Epidemiology & Data Science, Amsterdam UMC, Amsterdam, The Netherlands;* [9]*National Institute for Health Research Birmingham Biomedical Research Centre, Centre of Liver and Gastrointestinal Research, Institute of Immunology and Immunotherapy, University of Birmingham, Birmingham, UK;* [10]*Liver Unit, University Hospitals Birmingham National Health Service Foundation Trust Queen Elizabeth, Birmingham, UK;* [11]*Gastroenterology Hepatology and Transplantation Unit, ASST Papa Giovanni XXIII, Bergamo, Italy;* [12]*Department of Radiology, Fondazione IRCCS San Gerardo dei Tintori, Monza, Italy;* [13]*Translational Gastroenterology and Liver Unit, John Radcliffe Hospital and Nuffield Department of Medicine, University of Oxford, Oxford, UK;* [14]*Radcliffe Department of Medicine, University of Oxford, Oxford, UK*

Abbreviations

AIH, autoimmune hepatitis; ALP, alkaline phosphatase; AOM, Amsterdam–Oxford Model; AST, aspartate aminotransferase; CD, Crohn's disease; CRC, colorectal carcinoma; IBD, inflammatory bowel disease; IBD-U, IBD-unclassified; IPSCSG, International Primary Sclerosing Cholangitis Study Group; LASSO, Least Absolute Shrinkage and Selection Operator; LLN, lower limit of normal; LT, liver transplantation; MRCP, magnetic resonance cholangiopancreatography; MRI, magnetic resonance imaging; PRESTO, Primary Sclerosing Cholangitis Risk Estimate Tool; PSC, primary sclerosing cholangitis; qmAOM, quantitative MRCP-augmented Amsterdam–Oxford Model; ROC, receiver operating characteristic curve; UC, ulcerative colitis; ULN, upper limit of normal.

Financial support

This study was supported by a grant from Perspectum Limited. The company provided all MRCP+ data and had an advisory role, but no insight in the metadata.

Conflicts of interest

CYP received grants from NGM, Takeda, and Perspectum, and consultancy fees from Chemomab. ELC is on an advisory board and has a consulting role with Amgen, Zenus, Sanofi, Acepodia, Ipsen, Mirum, GSK, Dr Falk Pharma, and Gilead; received speaking fees from Dr Falk Pharma, Gilead, GSK, Mirum, Ipsen, and Amgen; and received institutional funding support from BRC Oxford NIHR (UK). MP is a shareholder in Perspectum Ltd. ELC and acknowledges support from the Oxford NIHR Biomedical Research Centre. TD and CF are employees of Perspectum Ltd.
Please refer to the accompanying ICMJE disclosure forms for further details.

Authors' contributions

Conceptualisation: TEM, LC, CYP. Methodology: TEM, LC, JAB, TD. Formal analysis: TEM, LC, JAB. Data curation and investigation: TEM, LC, WP, MT, SA-S, DD'A, PT, MP, CF. Supervision: MC, PT, MP, ELC, CYP. Writing – original draft: TEM, LC. Writing – review and editing: all authors. Reviewed and approved the final version of the manuscript: all authors.

Data availability

Data used in this study are not publicly available because of patient privacy, informed consent, and data-sharing agreements between participating centres. Anonymised data may be made available upon reasonable request and subject to approval by the participating centres and data-sharing agreements, and the relevant institutional review boards. Quantitative imaging metrics generated using the MRCP+ software (Perspectum Ltd., Oxford, UK) are subject to additional restrictions and cannot be shared publicly.

Supplementary data

Supplementary data to this article can be found online at https://doi.org/10.1016/j.jhepr.2026.101892.

References

Author names in bold designate shared co-first authorship

[1] Karlsen TH, Folseraas T, Thorburn D, et al. Primary sclerosing cholangitis - a comprehensive review. J Hepatol 2017;67:1298–1323.
[2] Boonstra K, Weersma RK, van Erpecum KJ, et al. Population-based epidemiology, malignancy risk, and outcome of primary sclerosing cholangitis. Hepatology 2013;58:2045–2055.
[3] Ponsioen CY, Chapman RW, Chazouilleres O, et al. Surrogate endpoints for clinical trials in primary sclerosing cholangitis: review and results from an International PSC Study Group consensus process. Hepatology 2016;63:1357–1367.
[4] Kim WR, Therneau TM, Wiesner RH, et al. A revised natural history model for primary sclerosing cholangitis. Mayo Clin Proc 2000;75:688–694.
[5] **de Vries EM**, **Wang J**, Williamson KD, et al. A novel prognostic model for transplant-free survival in primary sclerosing cholangitis. Gut 2018;67:1864–1869.
[6] Eaton JE, Vesterhus M, McCauley BM, et al. Primary Sclerosing Cholangitis Risk Estimate Tool (PREsTo) predicts outcomes of the disease: a derivation and validation study using machine learning. Hepatology 2020;71:214–224.
[7] Goode EC, Clark AB, Mells GF, et al. Factors associated with outcomes of patients with primary sclerosing cholangitis and development and validation of a risk scoring system. Hepatology 2019;69:2120–2135.
[8] Grigoriadis A, Imeen Ringe K, Bengtsson J, et al. Development of a prognostic MRCP-score (DiStrict) for individuals with large-duct primary sclerosing cholangitis. JHEP Rep 2022;4:100595.
[9] Ruiz A, Lemoinne S, Carrat F, et al. Radiologic course of primary sclerosing cholangitis: assessment by three-dimensional magnetic resonance cholangiography and predictive features of progression. Hepatology 2014;59:242–250.
[10] Vuppalanchi R, Are V, Telford A, et al. A composite score using quantitative magnetic resonance cholangiopancreatography predicts clinical outcomes in primary sclerosing cholangitis. JHEP Rep 2023;5:100834.
[11] Goldfinger MH, Ridgway GR, Ferreira C, et al. Quantitative MRCP imaging: accuracy, repeatability, reproducibility, and cohort-derived normative ranges. J Magn Reson Imaging 2020;52:807–820.
[12] Cazzagon N, El Mouhadi S, Vanderbecq Q, et al. Quantitative magnetic resonance cholangiopancreatography metrics are associated with disease severity and outcomes in people with primary sclerosing cholangitis. JHEP Rep 2022;4:100577.
[13] Cristoferi L, Porta M, Bernasconi DP, et al. A quantitative MRCP-derived score for medium-term outcome prediction in primary sclerosing cholangitis. Dig Liver Dis 2023;55:373–380.
[14] Ismail MF, Hirschfield GM, Hansen B, et al. Evaluation of quantitative MRCP (MRCP+) for risk stratification of primary sclerosing cholangitis: comparison with morphological MRCP, MR elastography, and biochemical risk scores. Eur Radiol 2022;32:67–77.
[15] Trivedi PJ, Arndtz K, Abbas N, et al. Quantitative MRCP and metrics of bile duct disease over time in patients with primary sclerosing cholangitis: a prospective study. Aliment Pharmacol Ther 2024;59:1366–1375.
[16] Middelburg TE, Mol B, Ferreira C, et al. Validation of quantitative magnetic resonance cholangiopancreatography metrics in prediction of transplant-free survival in primary sclerosing cholangitis. J Clin Exp Hepatol 2026;16:103417.
[17] Selvaraj EA, Ba-Ssalamah A, Poetter-Lang S, et al. A quantitative magnetic resonance cholangiopancreatography metric of intrahepatic biliary dilatation severity detects high-risk primary sclerosing cholangitis. Hepatol Commun 2022;6:795–808.
[18] Ponsioen CY, Assis DN, Boberg KM, et al. Defining primary sclerosing cholangitis: results from an international primary sclerosing cholangitis study group consensus process. Gastroenterology 2021;161:1764.75.e1765.
[19] European Association for the Study of the Liver. EASL clinical practice guidelines on sclerosing cholangitis. J Hepatol 2022;77:761–806.
[20] Chapman MH, Thorburn D, Hirschfield GM, et al. British Society of Gastroenterology and UK-PSC guidelines for the diagnosis and management of primary sclerosing cholangitis. Gut 2019;68:1356–1378.
[21] Bowlus CL, Arrive L, Bergquist A, et al. AASLD practice guidance on primary sclerosing cholangitis and cholangiocarcinoma. Hepatology 2023;77:659–702.
[22] Harisinghani M, Davis T, Ralli G, et al. Accuracy, repeatability, reproducibility and reference ranges of primary sclerosing cholangitis specific biomarkers from quantitative MRCP. Abdom Radiol (NY) 2025;50:5211–5222.
[23] Tibshirani R. Regression shrinkage and selection via the Lasso. J Roy Stat Soc B 1996;58:267–288.
[24] Simon N, Friedman J, Hastie T, et al. Regularization paths for Cox's proportional hazards model via coordinate descent. J Stat Softw 2011;39:1–13.
[25] Hothorn T, Lausen B. On the exact distribution of maximally selected rank statistics. Comput Stat Data Anal 2003;43:121–137.
[26] Graf E, Schmoor C, Sauerbrei W, et al. Assessment and comparison of prognostic classification schemes for survival data. Stat Med 1999;18:2529–2545.
[27] van Smeden M, Reitsma JB, Riley RD, et al. Clinical prediction models: diagnosis versus prognosis. J Clin Epidemiol 2021;132:142–145.
[28] Calendar year 2026 hospital outpatient prospective payment system (OPPS) and ambulatory surgical center final rule (CMS-1834-FC). https://www.federalregister.gov/documents/current.
[29] Shumbayawonda E, Bajre M, Eadle D, et al. Quantitative MRCP as part of primary sclerosing cholangitis standard of care in the National Health Service in England: a feasibility assessment among hepatologists. Healthcare (Basel) 2025;13:2630.

Keywords: Prognostication; Primary sclerosing cholangitis; Risk score; Quantification; Magnetic resonance cholangiopancreatography.
Received 5 November 2025; received in revised form 1 May 2026; accepted 5 May 2026; Available online 12 May 2026

Journal of Hepatology, Volume *8*

Supplemental information

Quantitative magnetic resonance cholangiopancreatography metrics improve prognostication in primary sclerosing cholangitis

Tim E. Middelburg, Laura Cristoferi, Willemijn Ponsioen, Maud Turkenburg, Carlos Ferreira, Tom Davis, Karin Horsthuis, Ynte S. de Boer, Adriaan J. van der Meer, Annemarie C. de Vries, Roy S. Dwarkasing, Johannes A. Bogaards, Sarah Al-Shakhshir, Palak Trivedi, Daphne D'Amato, Mauro Vigano, Eugenia V. Pesatori, Cesare Maino, Marco Carbone, Michael Pavlides, Jaap Stoker, Emma L. Culver, and Cyriel Y. Ponsioen

Quantitative magnetic resonance cholangiopancreatography metrics improve prognostication in primary sclerosing cholangitis

Tim E. Middelburg, Laura Cristoferi, Willemijn Ponsioen, Maud Turkenburg, Carlos Ferreira, Tom Davis, Karin Horsthuis, Ynte S. de Boer, Adriaan J van der Meer, Annemarie C. de Vries, Roy S. Dwarkasing, Johannes A. Bogaards, Sarah Al-Shakhshir, Palak Trivedi, Daphne D'Amato, Mauro Vigano, Eugenia V Pesatori, Cesare Maino, Marco Carbone, Michael Pavlides, Jaap Stoker, Emma L. Culver, Cyriel Y Ponsioen

Table of contents

Fig. S1 2
Fig. S2 3
Fig. S3 4
Fig. S4 5
Fig. S5 6
Fig. S6 7
Table S1 8
Table S2 9
Table S3 10
Table S4 11
Table S5 12
Tabel S6 12
Table S7 14
Table S8 15

Fig. S1 - Time dependent Area under the Curve for event-free survival by qmAOM score over 10 years time. Each line portrays the corresponding year.

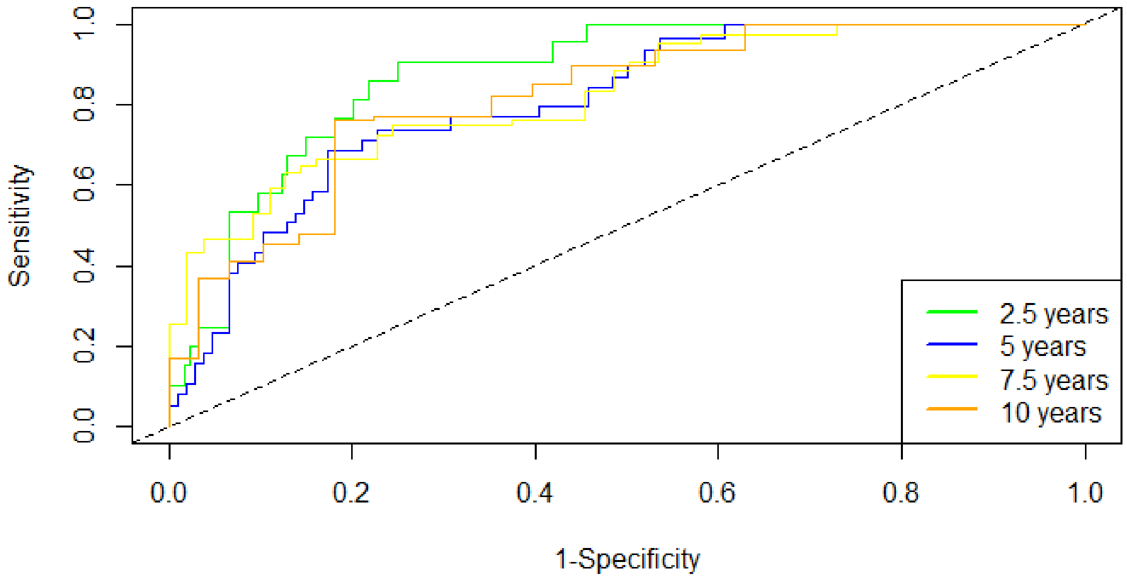

Time (years)	Cases	Survivors	Censored	AUC (%)
2.5	21	195	8	88.2%
5.0	38	114	72	81.6%
7.5	51	64	109	84.2%
10.0	61	122	141	83.6%

Fig. S2 - Kaplan-Meier curve of predicted survival versus observed survival over 10 years time. The black line portrays the observed survival, the blue line portrays the predicted survival.

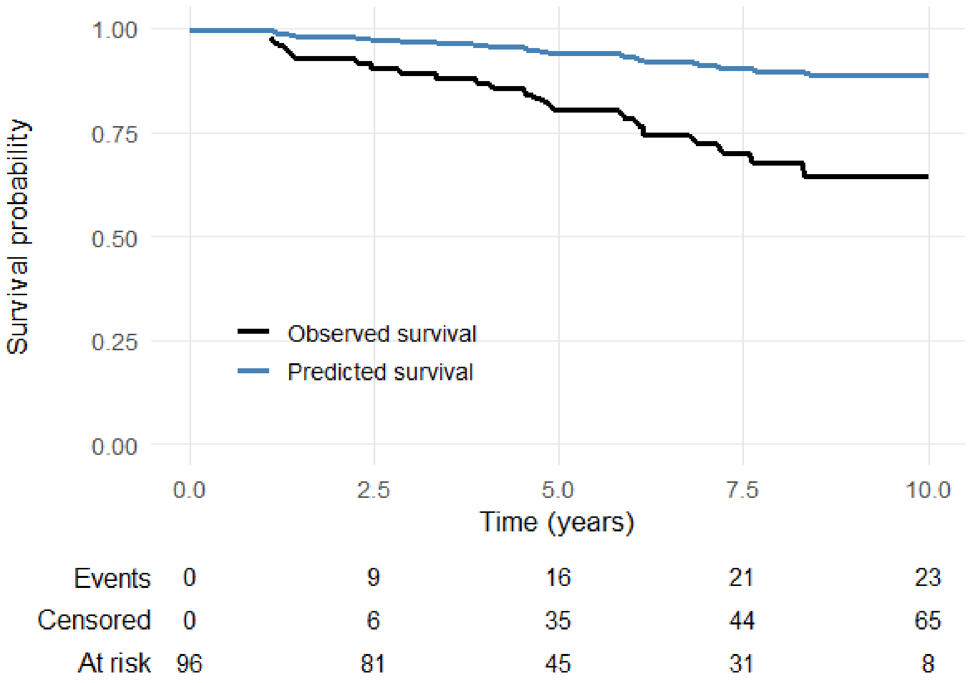

Fig. S3 - Kaplan-Meier curves risk groups based on the qmAOM score in the ANALI subset. Based on ≤ 1.29 as low-risk and > 1.29 as high-risk group.

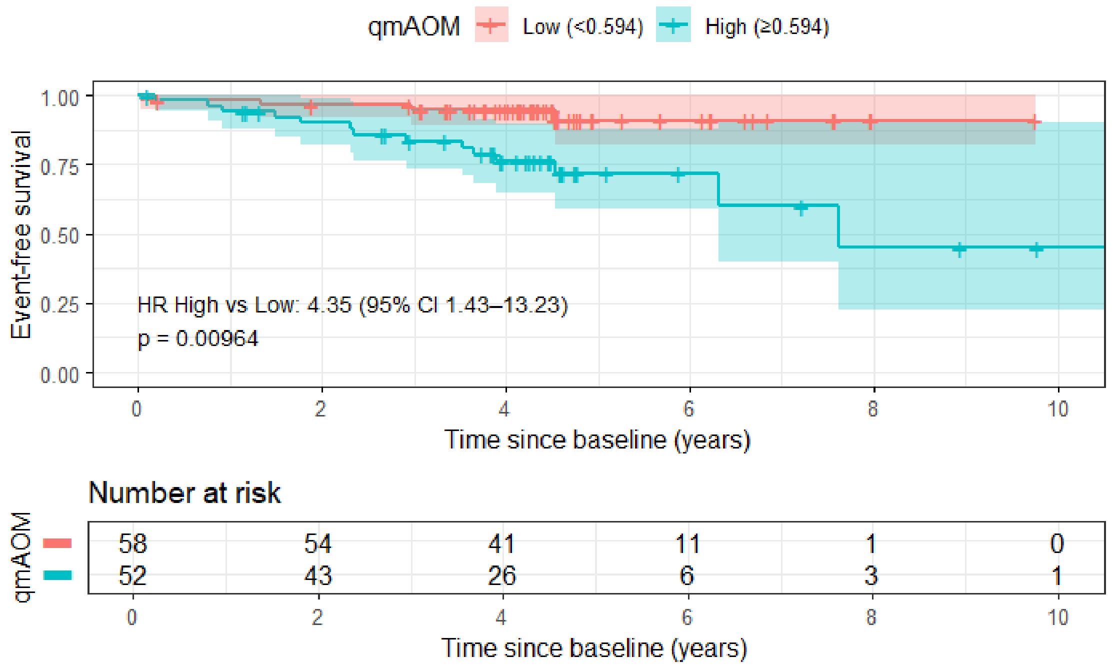

Fig. S4 - Kaplan-Meier curves of risk groups based on the total score of ANALI without gadolinium in the ANALI subset, including reviewer from Monza/Bergamo and reviewer 1 from Oxford. Based on ≤2 as low-risk and >2 as high-risk group.

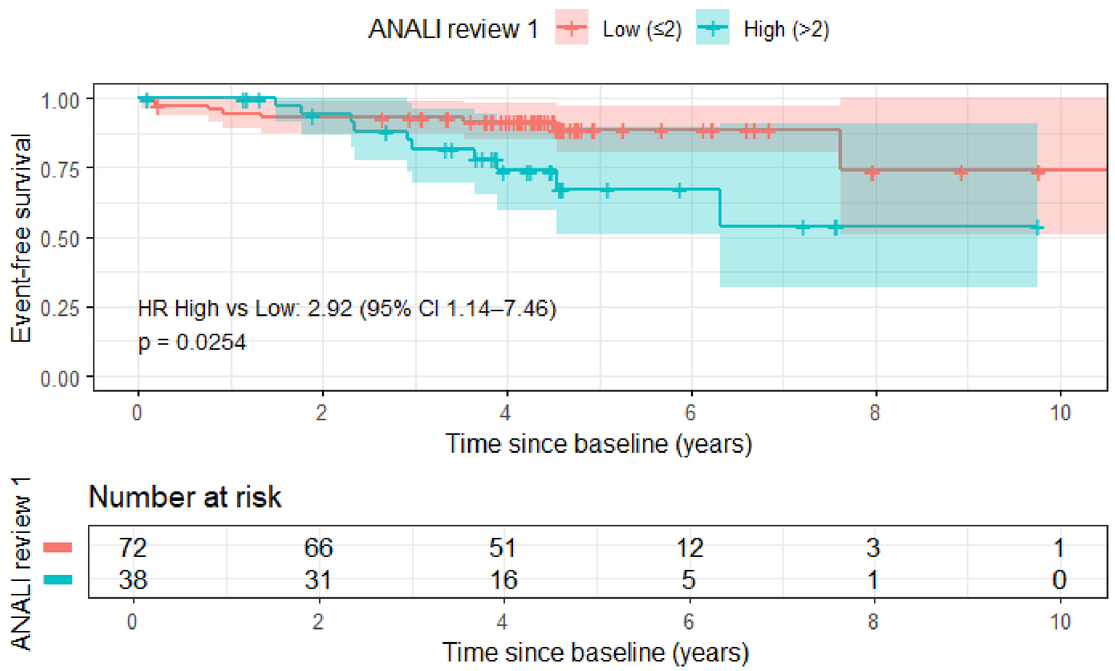

Fig. S5 - Kaplan-Meier curves of risk groups based on the total score of ANALI without gadolinium in the ANALI subset, including reviewer from Monza/Bergamo and reviewer 2 from Oxford. Based on ≤2 as low-risk and >2 as high-risk group.

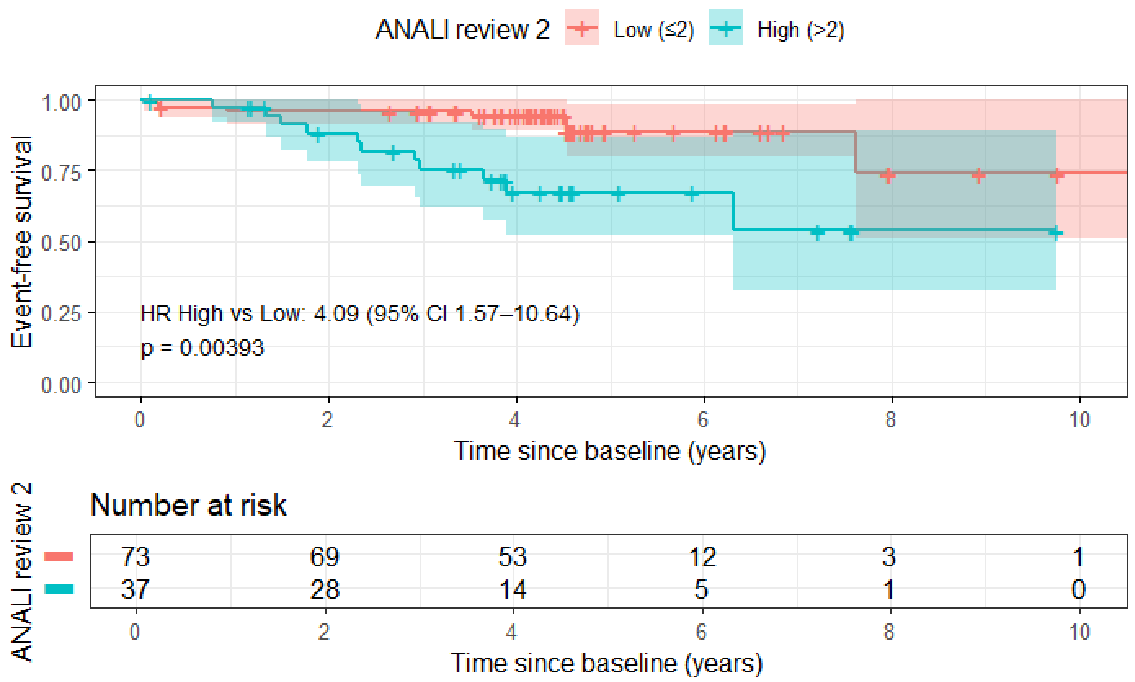

Fig. S6 - Heatmap of significant correlations between MRCP+ metrics, qmAOM and ANALI without gadolinium in the ANALI subset. Including components

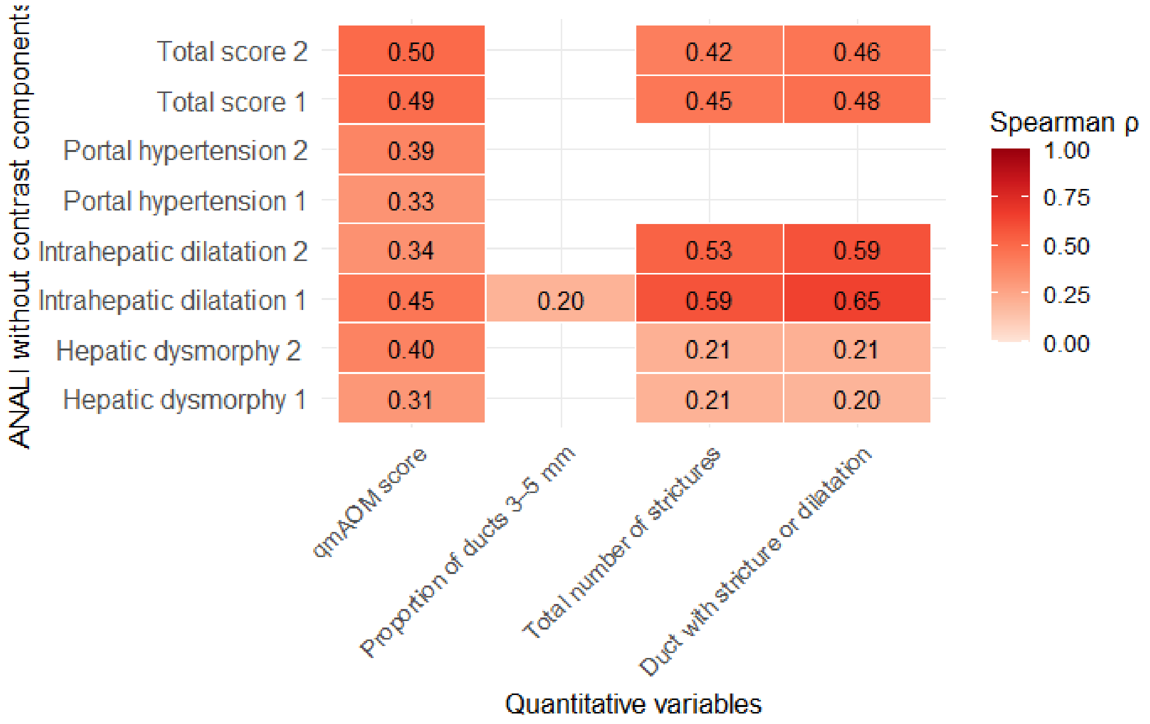

Table S1 - Cohort designs per participating centre

	Centre			
	Monza & Bergamo	***Birmingham***	***Oxford***	***Amsterdam & Rotterdam***
Study design	Retrospective	Prospective	Prospective	Retrospective
Minimum age of participants	> 16 years	> 18 years	> 18 years	> 18 years
PSC definition	EASL	UK-PSC, EASL	AASLD	IPSCSG
Minimal follow-up required	> 3 months	Not applicable	Not applicable	> 1 year
Fasting protocol	6-hours	4-hours	4-hours	Not specified*
Tesla strength	1.5T & 3T	3T	3T	1.5T & 3T

** Details on the fasting protocols were not retrievable, but routinely 4 hr fasting is prescribed before scan acquisition in the Netherlands*

Table S2 - MRI parameters per participants centre

	Centre			
Parameter	***Monza & Bergamo***	***Birmingham***	***Oxford***	***Amsterdam & Rotterdam***
Sequence	3D multi-shot fast/turbo spin echo	3D multi-shot fast/turbo spin echo	3D multi-shot fast/turbo spin echo	3D multi-shot fast/turbo spin echo
Plane	Coronal	Coronal	Coronal	Coronal
Triggering	Free breathing, navigator based	Free breathing, navigator based	Free breathing, navigator based	Free breathing, navigator based
Number of slices	72-192	72	60	40-240
Slice thickness, mm	1-2.5	1.1	1.1	1 – 2.20
Echo time (TE), ms	505-1144	708	604	441-1182
Repetition time (TR), ms	Breathing cycle	Breathing cycle	Breathing cycle	Breathing cycle
Acquisition matrix	256-416 x 200-356	258 x 320	256 x 256	236-448 x 181x387
FOV, mm	260-420 x 260-420	400x400	280	250-450 x 250-450
Flip angle (degrees)	90-140	120	120	90-140
iPAT factor	Yes	Yes	Yes	Yes
Number of averages	0.5-2	2	1.4	1-2
Fat suppression	Yes	Yes	Yes	Yes

Table S3 - Variable selection frequency by LASSO analysis in bootstrap framework

Variable	Selection frequency (%)
Serum albumin	99.6%
Serum total bilirubin	99.4%
Platelets	94.6%
Percentage of ducts with a 3-5mm median diameter	91.8%
Serum ALP	90.2%
IBD (yes/no)	70.4%
Centre type (transplantation/tertiary)	68.4%
Number of ducts with strictures or dilatations	62.8%
Age at PSC diagnosis	54.8%
Serum AST	52.2%
Total number of strictures	51.6%
Gender	46.6%
Sum of stricture length	42.8%
Median duct diameter	36.6%
Time from diagnosis to MRCP	35.2%
Serum ALT	27.4%
Age at MRCP, y	23.8%

mm: millimetre: ALP: Alkaline Phosphatase; IBD: inflammatory bowel disease; PSC: Primary sclerosing cholangitis; AST: Aspartate aminotransferase; MRCP: Magnetic resonance cholangiopancreatography; ALT: Alanine Aminotransferase

AST, ALP and total bilirubin are expressed in x the ULN and transformed to log-scale; Albumin and platelets are expressed as x the LLN and transformed to log-scale.

Table S4 - Patient demographics per centre type

		Tertiary care centre<br>*N=225*	Transplant centre<br>*N=232*	Overall<br>*N=457*
**Sex**	*Male*	145 (64.4%)	149 (64.2%)	294 (64.3%)
**Age at PSC diagnosis**, *y*		35.9 [26.1, 47.1]	36.8 [24.3, 47.9]	36.2 [25.2, 47.6]
**Type of PSC**	*Large Duct*	203 (90.2%)	223 (96.1%)	426 (93.2%)
	Small Duct	22 (9.8%)	9 (3.9%)	31 (6.8%)
**Type of IBD**	*None*	97 (43.1%)	99 (42.7%)	196 (42.9%)
	UC	113 (50.2%)	115 (49.6%)	228 (49.9%)
	CD	8 (3.6%)	17 (7.3%)	25 (5.5%)
	IBDu	7 (3.1%)	1 (0.4%)	8 (1.8%)
**Age at MRCP+**, *y*		44.0 [32.0, 54.0]	46.0 [29.8, 55.0]	45.0 [31.0, 54.0]
**PSC diagnosis to MRCP+**, *y*		6.0 [0.6, 11.3]	5.2 [2.0, 10.0]	5.6 [1.1, 10.6]
**MRCP+ to event or censoring**, *y*		4.8 [4.2, 8.5]	5.0 [2.2, 7.7]	4.9 [3.4, 8.0]
**PSC diagnosis to last follow-up**, *y*		12.0 [7.7, 17.5]	10.8 [6.8, 15.9]	11.4 [7.1, 16.6]
**ALP (U/L)**, *n = 429*		184 [115, 314]	215 [104, 368]	201 [111, 348]
**Bilirubin (umol/L)**, *n = 418*		13 [9, 21]	15 [10, 32]	14 [9, 25]
**Albumin (g/L)**, *n = 386*		41 [38, 44]$^{+}$	43 [39, 46]$^{+}$	42 [38, 45]
**AST (U/L)**, *n = 430*		45 [29, 86]	52 [31, 86]	49 [30, 86]
**Platelets (x10^9/L)**, *n = 414*		264 [196, 329]	245 [192, 299]	254 [194, 315]
**AOM risk score**, *n = 364*		1.6 [1.2, 2.0]	1.6 [1.2, 2.3]	1.6 [1.2, 2.1]
**AOM low risk (< 2.00)**, *n = 364*		139 (73.5%)	118 (67%)	257 (56.2%)
**Events**	***Primary endpoint****			
	Hepatic decompensation	44 (19.6%)	40 (17.2%)	84 (18.4%)
	Liver transplantation	13 (5.8%)	19 (8.2%)	22 (4.8%)
	PSCR-death exl. CRC	8 (3.6%)	25 (10.8%)	33 (7.2%)
	Secondary endpoints			
	PSCR-death or LT incl. CRC	48 (21.3%)	75 (32.3%)	123 (26.9%)
	PSCR-death or LT excl. CRC	48 (21.3%)	74 (32.9%)	122 (26.7%)

PSC: Primary sclerosing cholangitis; y: years; IBD: inflammatory bowel disease; UC; Ulcerative colitis; CD: Crohn's Disease: IBDu: unspecified IBD; MRCP: Magnetic resonance cholangiopancreatography; ALP: Alkaline Phosphatase; n: number of participants; AST: Aspartate aminotransferase; AOM: Amsterdam Oxford Model. PSCR: PSC-related. LT: Liver transplantation.
Reference ranges: ALP ≤ 120 U/L; Bilirubin ≤ 18 umol/L; Albumin ≥ 35 g/L; AST ≤ 35 U/L; Platelets ≥ 150 x 10^9/L.
Continuous numbers are displayed as total (%) or median [Q1, Q3]
** The composite primary endpoint shows which event occurred first in follow-up after MRCP+.*
$^{+}$ p-value < 0.01. The rest was non-significant.

Tabel S5 - Demographics in the derivation and validation set

		Derivation<br>*N=224*	Validation<br>*N=96*	Overall<br>*N=320*
Sex	*Male*	147 (65.6%)	60 (62.5%)	207 (64.7%)
Age at PSC diagnosis, *y*		35.0 [23.5, 47.0]	35.5 [26.7, 48.3]	35.0 [24.6, 47.4]
Type of PSC	*Large Duct*	210 (93.8%)	89 (92.7%)	299 (93.4%)
	Small Duct	14 (6.3%)	7 (7.3%)	21 (6.6%)
Type of IBD	*None*	87 (38.8%)	49 (51.0%)	136 (42.5%)
	UC	116 (51.8%)	44 (45.8%)	160 (50.0%)
	CD	15 (6.7%)	2 (2.1%)	17 (5.3%)
	IBDu	6 (2.7%)	1 (1.0%)	7 (2.2%)
Age at MRCP+, *y*		44.0 [31.8, 54.0]	47.0 [31.0, 56.0]	44.5 [31.0, 54.2]
PSC diagnosis to MRCP+, *y*		5.9 [2.0, 10.6]	4.5 [1.3, 11.4]	5.7 [1.5, 11.2]
MRCP+ to event or censoring, *y*		5.3 [4.1, 7.8]	4.8 [3.9, 8.1]	5.1 [4.0, 7.9]
PSC diagnosis to last follow-up, *y*		11.9 [7.8, 16.8]	11.4 [7.1, 15.9]	11.5 [7.7, 16.5]
ALP (U/L)		193 [113, 311]	163 [96, 355]	184 [107, 324]
Bilirubin (umol/L)		13 [9, 21]	13 [9, 24]	13 [9, 21]
Albumin (g/L)		42 [39, 46]	42 [39, 45]	42 [39, 45]
AST (U/L)		48 [30, 81]	42 [27, 77]	46 [29, 81]
Platelets ($x10^9$/L)		255 [193, 314]	255 [190, 308]	255 [193, 314]
AOM risk score		1.5 [1.2, 2.0]	1.5 [1.2, 2.0]	1.5 [1.2, 2.0]
AOM low risk (< 2.00)		58 (25.9%)	25 (26.0%)	83 (25.9%)
Event	*Primary endpoint*	64 (28.6%)	24 (25%)	88 (27.5%)

PSC: Primary sclerosing cholangitis; y: years; IBD: inflammatory bowel disease; UC; Ulcerative colitis; CD: Crohn's Disease: IBDu: unspecified IBD; MRCP: Magnetic resonance cholangiopancreatography; ALP: Alkaline Phosphatase; n: number of participants; AST: Aspartate aminotransferase; AOM: Amsterdam Oxford Model. Reference ranges: ALP ≤ 120 U/L; Bilirubin ≤ 18 umol/L; Albumin ≥ 35 g/L; AST ≤ 35 U/L; Platelets ≥ 150 x 10^9/L. Continuous numbers are displayed as total (%) or median [Q1, Q3]

No significant differences were found between the derivation and validation set.

Table S6 – Observed versus predicted survival and corresponding Brier score in the validation set.

	Year			
	**2.5**	**5**	**7.5**	**10**
**Observed survival**	91%	81%	70%	64%
**Mean predicted survival**	97%	94%	90%	89%
**Brier score**	0.06	0.11	0.14	0.14

Table S7 - Time dependent AUC for qmAOM, AOM and M+BA.

Year	2.5	5	7.5	10
**qmAOM AUC (95% CI)**	0.88 (0.82-0.95)	0.83 (0.75-0.90)	0.86 (0.79-0.93)	0.81(0.69-0.92)
**AOM AUC (95% CI)**	0.77 (0.67-0.87)	0.76 (0.69-0.85)	0.75(0.65-0.85)	0.74(0.61-0.86)
**M+BA AUC (95% CI)**	0.78 (0.69-0.87)	0.75 (0.67-0.83)	0.75(0.66-0.84)	0.55(0.40-0.70)
**At Risk**	195	114	64	22
**Events**	21	38	51	61

AUC: Area under the curve; qmAOM: quantitative MRCP modified Amsterdam Oxford Model; CI: Confidence intervals; AOM: Amsterdam Oxford Model; M+BA: MRCP+ bilirubin and aspartate aminotransferase score.

Table S8 - Time dependent AUC for qmAOM and ANALI without contrast as a subset analysis of all patients with available ANALI score data

Year	1	2	3	4	5
**qmAOM AUC (95% CI)**	0.73 (0.51-0.95)	0.79 (0.62-0.95)	0.77 (0.63-0.91)	0.83 (0.70-0.95)	0.75 (0.58-0.92)
**ANALI_1 AUC (95% CI)**	0.51 (0.41-0.63)	0.66 (0.52-0.81)	0.77 (0.65-0.88)	0.80 (0.70-0.90)	0.75 (0.60-0.89)
**ANALI_2 AUC (95% CI)**	0.63 (0.46-0.81)	0.76 (0.63-0.90)	0.84 (0.74-0.93)	0.85 (0.76-0.93)	0.70 (0.55-0.87)
**At Risk**	104	97	89	67	21
**Events**	4	7	11	14	16

AUC: Area under the curve; qmAOM: quantitative MRCP modified Amsterdam Oxford Model; CI: Confidence intervals

ANALI_1: Combination of Oxford reviewer 1 and Monza/Bergamo reviewer

ANALI_2: Combination of Oxford reviewer 2 and Monza/Bergamo reviewer
